# Molecular evolution and transcriptional profile of GH3 and GH20 β-N-acetylglucosaminidases in the entomopathogenic fungus *Metarhizium anisopliae*

**DOI:** 10.1590/1678-4685-GMB-2017-0363

**Published:** 2018-12-10

**Authors:** Eder Silva de Oliveira, Ângela Junges, Nicolau Sbaraini, Fábio Carrer Andreis, Claudia Elizabeth Thompson, Charley Christian Staats, Augusto Schrank

**Affiliations:** ^1^ Centro de Biotecnologia, Universidade Federal do Rio Grande do Sul, Porto Alegre, RS, Brazil

**Keywords:** NAGases, GH20 and GH3, Metarhizium, chitinolytic system, entomopathogenesis

## Abstract

Cell walls are involved in manifold aspects of fungi maintenance. For several fungi, chitin synthesis, degradation and recycling are essential processes required for cell wall biogenesis; notably, the activity of β-N-acetylglucosaminidases (NAGases) must be present for chitin utilization. For entomopathogenic fungi, such as *Metarhizium anisopliae*, chitin degradation is also used to breach the host cuticle during infection. In view of the putative role of NAGases as virulence factors, this study explored the transcriptional profile and evolution of putative GH20 NAGases (*MaNAG1* and *MaNAG2*) and GH3 NAGases (*MaNAG3* and *MaNAG4*) identified in *M. anisopliae*. While MaNAG2 orthologs are conserved in several ascomycetes, MaNAG1 clusters only with *Aspergilllus* sp. and entomopathogenic fungal species. By contrast, MaNAG3 and MaNAG4 were phylogenetically related with bacterial GH3 NAGases. The transcriptional profiles of *M. anisopliae* NAGase genes were evaluated in seven culture conditions showing no common regulatory patterns, suggesting that these enzymes may have specific roles during the *Metarhizium* life cycle. Moreover, the expression of *MaNAG3* and *MaNAG4* regulated by chitinous substrates is the first evidence of the involvement of putative GH3 NAGases in physiological cell processes in entomopathogens, indicating their potential influence on cell differentiation during the *M. anisopliae* life cycle.

## Introduction

Chitin is the second most abundant polymer on Earth and its recycling from carapaces, cuticles and fungal cell walls impacts on carbon and nitrogen cycles. The chitin polymer is composed of β-1,4-linked N-acetyl-D-glucosamine (GlcNAc) subunits (Beier and Bertilsson, 2013) and its degradation can be driven in two ways: i) chitin can be deacetylated to chitosan by action of chitin deacetylases (EC 3.5.1.41), which yields glucosamine monomers via the enzymatic hydrolysis by chitosanase (EC 3.2.1.132); or ii) by the chitinolytic degradation process generating GlcNAc monomers, which involves the initial hydrolysis of the β-1,4 glycoside bonds by the action of a group of enzymes, including chitinases (EC 3.2.1.14), lytic polysaccharide monooxygenases (LPMOs) (of the auxiliary activity 10 family - AA10; EC N/A) and β-N-acetylglucosaminidases (NAGases; EC 3.2.1.52) (Beier and Bertilsson, 2013; Thorat *et al.*, 2017). The enzymes evolved in the chitinolytic degradation process act in a consecutive fashion to completely degrade chitin (Patil *et al.*, 2000; Hartl *et al.*, 2012; Brzezinska *et al.*, 2014). LPMOs and endo-acting GH18 chitinases insert strand breaks at random positions within the chitin polymer, while exo-acting GH18 chitinases subsequently cleave chito-oligosaccharides (Chavan and Deshpande, 2013; Langner and Göhre, 2015). Finally, NAGases hydrolyze β-1,4 linkages on N-acetylglucosamine dimers (chitobiose), producing GlcNAc monosaccharides (Duo-Chuan, 2006).

NAGases are classified into three glycoside hydrolase (GH) families, 3, 20, and 84, on the basis of their amino acid sequence similarities (Cantarel *et al.*, 2009). GH3 and GH84 NAGases are distributed in several bacterial and metazoan cells, respectively, while members from the GH20 family are versatile enzymes abundant in fungi and insects. Although these three families encompass functionally related enzymes, they possess no sequence homology, differing in their structure and catalytic mechanism (Slámová *et al.*, 2010; Liu *et al.*, 2012).

The genomes of ascomycetous filamentous fungi contain, on average, 15 to 25 chitinase-encoding genes, but only one or two genes encoding GH20 NAGases (Seidl, 2008; Junges *et al.*, 2014). Notably, as has been shown for the mycopathogenic fungus *Trichoderma atroviride*, chitin could not be used as a nutrient source if NAGase activity is absent, despite the presence of approximately 30 chitinase genes, emphasizing the importance of these enzymes for the full degradation of the chitin polymer (López-Mondéjar *et al.*, 2009). In this way, the diversity of chitinase genes contrasts with the relatively low number of NAGase genes and their fundamental importance on chitin metabolism.

Potential functions for NAGases in fungi include the use of exogenous chitin as a nutrient source and cell wall turnover during the fungal life cycle (Seidl *et al.*, 2006). These functions have already been described for GH20 NAGases in *T. atroviride* (Seidl *et al.*, 2006; López-Mondéjar *et al.*, 2009), *Aspergillus* sp*.* (Kim *et al.*, 2002), and *Neurospora crassa* (Tzelepis *et al.*, 2012). In addition, GH20 NAGases participate in processes related to fungal hyphal extension and branching (Rast *et al.*, 1991), fungal cell wall degradation during autolysis (Díez *et al.*, 2005), and have a putative role in insect pathogenesis (St. Leger *et al.*, 1991).

In contrast, NAGases belonging to the GH3 family consist of a small group of bacterial enzymes that possess a broad range of functions depending on the organism. Similarly to GH20 NAGases, some GH3 NAGases participate in chitin catabolism, as in marine chitinolytic bacteria, such as *Vibrio furnissii* and *Alteromonas* sp. (Tsujibo *et al.*, 1994; Chitlaru and Roseman, 1996). Notably, only recently, the first fungal GH3 NAGase was described (Yang *et al.*, 2014). The RmNag enzyme from the zygomycete *Rhizomucor miehei* exhibited hydrolysis activity on N-acetylchitooligosaccharide (GlcNAc)_2-3_ substrates. This report further supports the existence of GH3 NAGases in other fungal species, especially in ascomycetes, considering their expansion of chitinolytic machinery genes (Seidl 2008; Junges *et al.*, 2014).

In recent years the chitin degradation machinery has attracted much attention, especially in entomopathogenic fungi, such as *Metarhizium anisopliae* (Hypocreales: Clavicipitaceae). In these species, the chitinolytic system has, probably, two main biological functions: Firstly, as chitin is the major component of fungal cell walls, chitin-degrading enzymes act on the cell wall remodeling, which is necessary for hyphal vegetative growth (Seidl, 2008). Secondly, the infection of arthropod hosts requires a prior chitin hydrolysis of the exoskeleton (St. Leger *et al.*, 1991). Furthermore, *M. anisopliae* has the ability to differentiate into specialized cell types during its infection cycle. The switch between conidia to hyphae and the formation of infection structures (i.e., appressorium and blastospore), are processes that require chitin degradation (Schrank and Vainstein, 2010). Notably, the importance of some *M. anisopliae* chitinase genes in infection process have been suggested and functionally verified using knockout constructions (da Silva *et al.*, 2005; Boldo *et al.*, 2009; Staats *et al.*, 2013).

Despite the knowledge gained by the study of chitinases in *Metarhizium*, the role of NAGases in the life cycle and infection process of entomopathogens has not been fully elucidated. This study surveyed putative NAGase genes from GH3 and GH20 families in *M. anisopliae* and investigated their evolutionary relationships to those of other filamentous ascomycetes. To further characterize NAGase genes in *M. anisopliae*, their expression patterns were evaluated in different cell types and various nutritional conditions. The results suggest new possibilities for studying NAGases participation in *M. anisopliae* biology.

## Material and Methods

### NAGase gene mining of the *M. anisopliae* genome

The survey of NAGase genes was performed in the *M. anisopliae* E6 genome assembly (accession number PRJNA245858) (Staats *et al.*, 2014). In order to identify putative GH20 NAGase genes, three well described NAGase sequences of filamentous fungi were used as the query in a tBLASTn search: NagA from *A. nidulans* (XP_659106) (Kim *et al.*, 2002), and Nag1 and Nag2 from *T. atroviride* (EHK40646 and EHK46127) (Brunner *et al.*, 2003; López-Mondéjar *et al.*, 2009). Further screening was performed using the conserved GH20 domain sequence found in GH20 hexosaminidases (InterProScan IPR015883) as the query. To identify *M. anisopliae* putative NAGases of the GH3 family, the NagA protein sequence from the bacteria *Streptomyces thermoviolaceus* OPC-520 (BAA32403) was used as a query in the tBLASTn search (Tsujibo *et al.*, 1998). Additionally, the GH3 RmNag sequence from the zygomycete *R. miehei* CAU-432 (AGC24356), the only fungal GH3 family member with NAGase activity to date (Yang *et al.*, 2014), was also used a query. Further screening was performed using the conserved GH3 domain sequence from GH3 hexosaminidases (InterProScan IPR001764) as a query. All NAGase sequences were extracted from the BROAD Institute and NCBI databases.

Each identified NAGase sequence was applied to search for similarity on *M. anisopliae* contigs employing the tBLASTn algorithm in the BioEdit software (Hall, 1999). The positive NAGase containing contigs were screened for GH20 and GH3 family domains. The same screening methodology was applied using the conserved sequence motif from GH20 NAGases (H/N-x-G-A/C/G/M-D-E-A/I/L/V) (Slámová *et al.*, 2010) and the conserved motif from GH3 NAGases (K-H-F/I-P-G-H/L-G-x-x-x-x-D-S/T-H) (Mayer *et al.*, 2006).

### NAGase sequence analyses

To further confirm and analyze the specific GH20 and GH3 NAGases domains identified by the *in silico* survey, the predicted sequences were compared with sequences deposited on InterProScan (Zdobnov and Apweiler, 2001), dbCAN (Yin *et al.*, 2012) and CDD (Conserved Domain database) databases (Marchler-Bauer *et al.*, 2009). Additionally, BLASTx and manual inspection (search for canonical 5’ and 3’ splice sites) was employed to predict and compare the number and position of introns between *M. anisopliae* putative NAGase gene sequences and public NAGase sequences. Theoretical isoelectric points and molecular mass values were obtained from Compute p*I*/Mw tool (Bjellqvist *et al.*, 1993, 1994). Transmembrane domains were investigated by TMHMM v.2.0 (Krogh *et al.*, 2001). Theoretical signal peptide cleavage sites were analyzed by the SignalP 4.1 server (Petersen *et al.*, 2011). GPI-anchoring signals were predicted by the big-PI Fungal Predictor software (Eisenhaber *et al.*, 2004). Non-classical secretion pathway prediction was evaluated by the SecretomeP server 2.0 (Bendtsen *et al.*, 2004) and the number of N-glycosylation sites was predicted by the GlycoEP Predictor (Chauhan *et al.*, 2013).

### NAGase protein phylogeny

*M. anisopliae* putative GH20 and GH3 NAGase sequences were employed to identify ortholog sequences in 15 filamentous fungi species (Table 1). RmNAG of the zygomycete *R. miehei* and 10 well described bacterial GH3 NAGases were added to the phylogenetic analysis of GH3 NAGases. Additionally, *M. anisopliae* β-glucosidases, characterized fungal β-glucosidases and putative β-glucosidases from species described in Table 1, were used as outgroup for the phylogenetic analysis.

**Table 1:** List of microorganisms used in GH20 and GH3 NAGases phylogenetic analysis.

Category^a^	Microorganisms	Protein name^b^	Reference
Fungi			
A, B, C	*Aspergillus fumigatus* Af293		(Nierman *et al*., 2005)
A, B, C	*Aspergillus nidulans* FGSC A4		(Galagan *et al*., 2005; Wortman *et al*., 2009)
A, B, C	*Aspergillus niger* CBS 513.88		(Pel *et al*., 2007)
A, B, C	*Beauveria bassiana* ARSEF 2860		(Xiao et al., 2012)
A, B, C	*Cordyceps militaris* CM01		(Zheng *et al*., 2011)
A, B, C	*Fusarium graminearum* PH-1		(Cuomo *et al*., 2007)
A, B, C	*Fusarium oxysporum* f. sp. *cubense*		(Guo *et al.*, 2014)
A, B, C	*Magnaporthe oryzae* 70-15		(Dean *et al*., 2005)
A, B, C	*Metarhizium acridum* CQMa 102		(Gao *et al*., 2011)
A, B, C	*Metarhizium robertsii* ARSEF 23		(Gao *et al*., 2011; Hu *et al*., 2014)
A, B, C	*Nectria haematococca* MPVI 77-13-4		(Coleman *et al*., 2009)
A, B	*Neurospora crassa* OR74A		(Galagan *et al*., 2003)
A, B, C	*Trichoderma atroviride* IMI 206040		(Kubicek *et al*., 2011)
A, B, C	*Trichoderma reesei* QM6a		(Martinez *et al*., 2008)
A, B, C	*Trichoderma virens* Gv29-8		(Kubicek *et al*., 2011)
B	*Rhizomucor miehei*	RmNag	(Yang *et al*., 2014)
C	*Amesia atrobrunnea*	CEL3a, CEL3b	(Colabardini *et al*., 2016)
C	*Aspergillus aculeatus*	BGL1	(Kawaguchi *et al*., 1996)
C	*Aspergillus oryzae* RIB40	BglA, BglF, BglJ	(Kudo *et al*., 2015)
C	*Neurospora crassa* OR74A	BGL2	(Pei *et al*., 2016)
C	*Penicillium brasilianum*	BGL1	(Krogh *et al*., 2010)
C	*Thermothelomyces thermophila* ATCC 42464	MtBgl3b	(Zhao *et al*., 2015)
C	*Ustilago esculenta*	UeBgl3A	(Nakajima *et al*., 2012)
Bacteria			
B	*Alteromonas sp.* 0-7	HEXA	(Tsujibo *et al*., 1994)
B	*Bacillus subtilis* 168	NAGZ	(Liu *et al*., 1997)
B	*Cellulomonas fimi*	NAG3	(Mayer *et al*., 2006)
B	*Clostridium paraputrificum* M-21	NAGZ	(Li *et al*., 2003)
B	*Escherichia coli* K-12	NAGZ	(Cheng *et al*., 2000)
B	*Streptomyces thermoviolaceus* OPC-520	NAGA	(Tsujibo *et al*., 1998)
B	*Thermotoga maritima* NSB-8	NAGA	(Choi *et al*., 2009)
B	*Thermotoga neapolitana* KCCM-41025	CBSA	(Choi *et al*., 2009)
B	*Vibrio cholerae*	NAGZ	(Stubbs *et al*., 2007; Balcewich *et al*., 2009)
B	*Vibrio furnissii* 7225	NAGZ	(Chitlaru and Roseman, 1996)

^a^ Microorganisms were classified according to their use in phylogenetic analysis: (A) microorganisms containing *M. anisopliae* GH20 NAGases orthologs; (B) microorganisms containing *M. anisopliae* GH3 NAGases orthologs; and (C) microorganisms containing β-glucosidases included as an outgroup in GH3 NAGase phylogenetic analysis.

^b^ Named proteins are characterized enzymes.

Only fungal sequences were used for the inference of the phylogenetic tree of GH20 NAGases, since alignment errors are more frequent when divergent sequences are included in the analysis. The amino acid alignments were built and trimmed with GUIDANCE2 (Sela *et al.*, 2015) using PRANK (Löytynoja and Goldman, 2010) as an MSA algorithm with 100 bootstrap replicates and the additional default parameters. The cut-off score for filtering unreliably aligned amino acids was chosen to be 0.60, after the multiple alignments were manually checked. The best-fit evolutionary model was evaluated using ProtTest 3.4 (Darriba *et al.*, 2011). MrBayes 3.2.5 (Ronquist *et al.*, 2012) and PhyML 3.1 (Guindon *et al.*, 2010) were used to infer the GH3 and GH20 NAGase phylogenetic trees using Bayesian inference (BI) and maximum likelihood (ML), respectively. Four chains were run for 1,000,000 generations, sampled every 100 steps, with an average standard deviation of split frequencies < 0.01 as convergence criterion and 25% of genealogies discarded as burn-in in the BI analysis. In the ML analysis, a fast approximate likelihood ratio test (aLRT) was used for determining the branch support, which is a an appropriate alternative for the computationally demanding bootstrap analysis (Anisimova and Gascuel, 2006; Anisimova *et al.*, 2011).

### Fungal strain and culture conditions

*Metarhizium anisopliae* E6 strain was isolated from the insect *Deois flavopicta* in Brazil. Conidia were collected from agar plate cultures and filtered with glass wool to remove the mycelium. *M. anisopliae* conidial suspensions (110^6^ conidia/mL) were cultured under seven different growth conditions prior to RNA extraction: i) Cove’s Complete medium (MCc) containing (w/v) 1% glucose, 0.6% NaNO_3_, 0.15% casein hydrolisate, 0.05% yeast extract, 0.2% peptone, pH 7.0 plus 2% (v/v) salts solution [2.6% KCl, 2.6% MgSO_4_.7H_2_O and 7.6% KH_2_PO_4_ (w/v)] and 0.04% (v/v) Trace Elements Solution [0.04% Na_2_Ba_4_O_7_.7H_2_O, 0.4% CuSO_4_.5H_2_O, 0.01% FeSO_4_, 0.8% Na_2_MNO_4_.7H_2_O, 0.8% MnSO_4_.7H_2_O and 0.8% ZnSO_4_.7H_2_O (w/v)] (Pinto *et al.*, 1997); ii) 0.25% GlcNAc in minimum medium composed of 0.6% NaNO_3_ (w/v) plus 0.25% GlcNAc) (w/v) as carbohydrate source, with salts and trace element solutions (Junges *et al.*, 2014); iii) 1% Chitin in minimum medium composed of 0.6% NaNO_3_ (w/v) plus 1% crystalline chitin from crab shells as a carbohydrate source, with salts and trace element solutions (Junges *et al.*, 2014). *M. anisopliae* cultures i, ii and iii were maintained on a shaker (180 rpm) for 72 h at 28 °C, then washed with sterile distilled water and filtered through *Miracloth* and frozen in liquid nitrogen for total RNA extraction; iv) Autolysis: medium for mycelium autolysis induction (1% glucose (w/v) and 0.6% NaNO_3_ (w/v), sustained for 9 days) (Junges *et al.*, 2014; Kappel *et al.*, 2016); v) Sporulation: on MCc agar plates for conidia RNA extraction; vi) Blastospores: Inoculation of 510^4^ conidia/mL on ADAMEK medium for blastospore production [3% corn steep solids, 4% glucose and 3% yeast extract (w/v)], shaking for 64 h at 28 ºC (Adamek, 1965); vii) Appressorium induction medium: 510^5^ conidia/mL was inoculated in 0.004% yeast extract solution on 500 glass coverslips for 16 h at 28 ºC (Junges *et al.*, 2014). Blastospore and appressorium induction were confirmed by microscopic observation of randomly selected coverslips (Figure S1).

### RNA sample preparation

Total RNA extraction from *M. anisopliae* cells harvested under all seven different growth conditions was performed in triplicate. Samples were ground using a mortar and pestle in liquid nitrogen, prior to standard RNA extraction using Trizol Reagent (Life Technologies, Grand Island, NY, USA). Residual DNA was removed with DNase (Thermo Scientific, MA, USA). Thereafter, extracted RNAs were passed through RNeasy Cleanup columns (Qiagen, Hilden, Germany). RNA samples were quantified using a Qubit fluorometer (Life Technologies, Grand Island, NY, USA), and stored at -80 °C. One microgram of total RNA was used for cDNA synthesis using MMLV-RT enzyme (Life Technologies, Grand Island, NY, USA). All procedures were performed according to the manufacturer’s instructions.

### Quantitative PCR (qPCR) experiments

Polymerase chain reactions were carried out on ABI-7500 Real-Time PCR System (Applied Biosystems, Foster City, CA, USA). Platinum SYBR Green qPCR SuperMix-UDG (Life Technologies, Grand Island, NY, USA) was used to monitor dsDNA synthesis. Each biological sample was analyzed in technical triplicates; no-template and no-reverse transcriptase controls were included.

Primers for qPCR assays were designed using VECTOR NTI software (Thermo Fisher Scientific, Waltham, MA, USA) (Table S1). Five housekeeping genes were evaluated: *act* (γ-actin), *gapdh* (glyceraldehyde 3-phosphate dehydrogenase), *tef1-*α (translation elongation factor 1-α), *trp1* (tryptophan biosynthesis enzyme), and *tub* (α-tubulin). The efficiency of each reference gene across samples was analyzed using *geNorm* version 3.5 (Vandesompele *et al.*, 2002) and *NormFinder* (Andersen *et al.*, 2004). The best reference gene identified by both analyses for the samples tested was *tef1-*α, which was subsequently used in all qPCR assays (Table S1).

Melting curves from each qPCR reaction were analyzed to confirm specificity of the synthesized products and absence of primer dimers. Relative transcript expressions were analyzed by Cq (quantification cycle) values, applying the 2^-DDCt^ method (Livak and Schmittgen, 2001). Results were processed in GraphPad Prism (La Jolla, CA, USA) for graphics and statistical data acquisition. One-way analysis of variance (ANOVA), followed by Tukey’s multiple comparisons test (*p* < 0.05) were performed to determine statistical differences among 2^-DDCt^ values of the seven experimental conditions.

## Results

### *M. anisopliae* putative GH20 and GH3 NAGases

**Figure 1:**
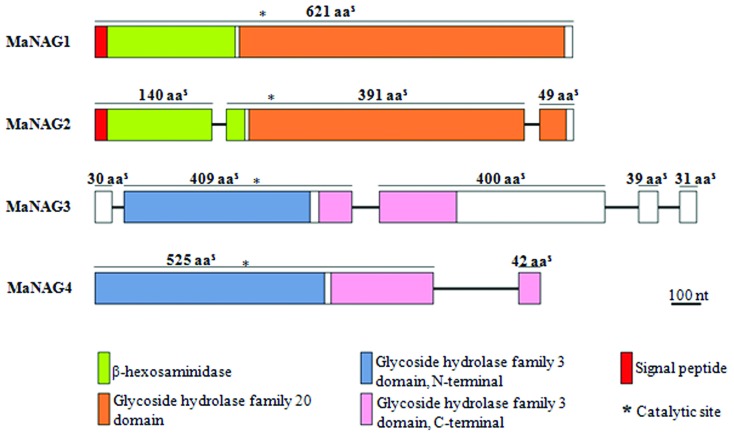
Modular domain structure from *M. anisopliae* NAGase genes. NAGase genes exhibit specific conserved domains with different compositions. Coding exonic sequences are depicted as boxes (color codes are indicated) and introns as thin lines. Domains were identified using Conserved Database Domain (at NCBI), dbCAN and InterProScan. Signal peptide sequences were predicted using SignalP 4.1. Blank protein regions indicate the absence of characterized domains.

The survey of NAGase genes of the *M. anisopliae* genome, using NagA from *A. nidulans* and NAG1 and NAG2 from *T. atroviride* as queries, resulted in the identification of two putative GH20 NAGases, named MaNAG1 (MANI_010908; GenBank accession number KFG80340) and MaNAG2 (MANI_029504; GenBank accession number KFG85702). All other fungal GH20 NAGase sequences and GH20 conserved domain sequences used as queries resulted in alignments with the same two previously detected contigs. Therefore, MaNAG1 and MaNAG2 are most probably the only *M. anisopliae* putative GH20 NAGases. The GH20 family domain (IPR015883) and the conserved motif of GH20 proteins (H/N-x-G-A/C/G/M-D-E-A/I/L/V) were found in both MaNAG1 and MaNAG2 sequences (Figure S2). Additionally, the putative GH20 NAGases also exhibited a chitobiase/beta-hexosaminidase N-terminal domain (IPR029018) (Figure 1).

**Figure 2:**
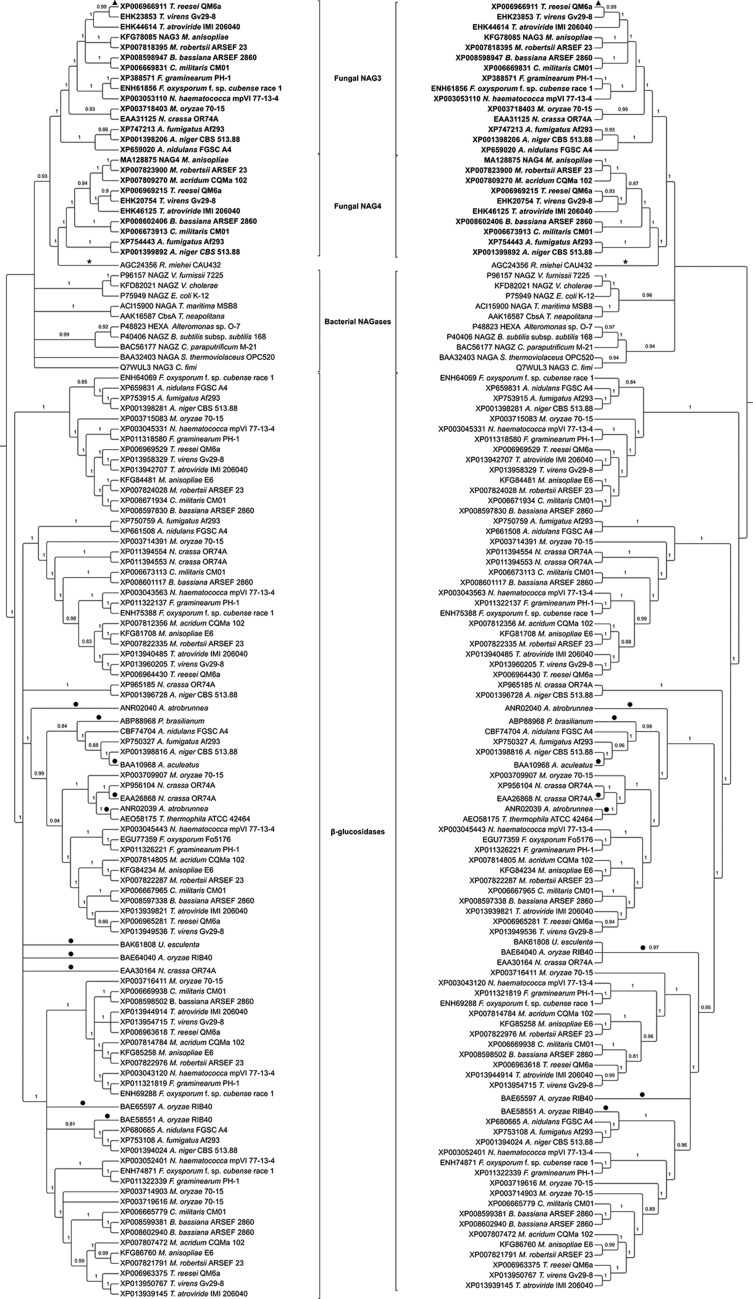
Phylogenetic relationships among GH3 NAGases from filamentous fungi, bacteria and zygomycetes. Putative and characterized fungal GH3 β-glucosidases were included as an outgroup. The phylogenies were obtained using MrBayes 3.2.5 (left side) and PhyML 3.1 (right side). ★: NAGase from the zygomycete*Rhizomucor miehei*. ●: Nodes with support values below 0.8 were collapsed into polytomies.

The GH3 domain screening of the *M. anisopliae* genome allowed the identification of seven positive matches. However, phylogenetic analysis clearly revealed that only two sequences, named MaNAG3 (MANI_122030; GenBank accession number KFG78085) and MaNAG4 [MANI_128875; (Figure S3)] could be putative GH3 NAGases. Furthermore, these sequences exhibit higher similarity with bacterial GH3 NAGases and the RmNag GH3 (Figure S4). MaNAG3 and MaNAG4 share a conserved domain with GH3 family members (IPR001764) and exhibit the conserved sequence motif of GH3 proteins (K-H-F/I-P-G-H/L-G-x-x-x-x-D-S/T-H) (Figure S4). Furthermore, MaNAG3 and MaNAG4 sequences present a conserved GH3 C-terminal domain (IPR002772) (Figure 1). The other five putative GH3 proteins (KFG84234, KFG86760, KFG85258, KFG81708, and KFG84481) display higher sequence conservation and are phylogenetic related with fungal β-glucosidases (Figure 2 and Figure S4), raising the possibility of functional equivalence.

All properties of the proposed *M. anisopliae* putative NAGases are listed in Table 2. Putative GH20 NAGase genes have similar ORF sizes and exhibit no intron conservation between sequences. While MaNAG1 does not show any intron insertions, the MaNAG2 sequence has two intron insertions (Figure 1). The predicted molecular masses for MaNAG1 and MaNAG2 (66.98 kDa and 61.42 kDa, respectively) are similar to other fungal GH20 NAGases, *A. nidulans* NagA (68 kDa) (Kim *et al.*, 2002), *T. atroviride* Nag1 (73 kDa) (Brunner *et al.*, 2003) and *T. harzianum* P1 Nag1 (72 kDa) (Peterbauer *et al.*, 1996).

**Table 2:** Properties of *Metarhizium anisopliae* GH20 and GH3 β-N-acetylglucosaminidases.

Identification	GH family	ORF length (nt)	Introns	Protein length (aa)	Mature protein theoretical kDa	Theoretical pI	Conserved domain	Transmembrane domain	Signal peptide	GPI or NCS	N-glycosylation site	Acession number
MaNAG1	GH20	1,863	0	620	66.98	6.07	cl02948 / pfam14845	-	+	-	4	KFG80340
MaNAG2	GH20	1,862	2	579	61.42	4.85	cd06562 / pfam14845	-	+	-	4	KFG85702
MaNAG3	GH3	2,730	4	909	98.71	6.25	COG1472	+	-	-	6	KFG78085
MaNAG4	GH3	1,701	1	566	60.67	5.6	COG1472	-	-	-	2	MANI_ 128875

(+): presence; (-) absence; pI: isoelectric point; GPI: GPI-anchor sites; NCS: non-classical secretion pathway regions.

The theoretical pI of *M. anisopliae* GH20 NAGases predicts that they are acidic enzymes, with MaNAG2 exhibiting a more acidic pI than MaNAG1, 4.85 and 6.07, respectively. Both putative GH20 NAGases have the same four N-glycosylation sites. Putative GH3 NAGase genes exhibit different physicochemical properties. MaNAG3 is the largest gene (3,223 bp), containing the highest expected number of introns (4) and theoretical molecular mass (98.71 kDa), with N-glycosylation translational modification signals on six sites. In contrast, MaNAG4 ORF size is 2,057 bp, the theoretical molecular mass is 60.67 kDa and the pI of predicted mature protein is 5.6. The predicted molecular mass of MaNAG4 is similar to most known bacterial GH3 NAGases, as *S. thermoviolaceus* NagA (60 kDa) (Tsujibo *et al.*, 1998). None of the putative NAGase protein sequences contain GPI-anchoring sites or non-classical secretion pathway prediction signals. Interestingly, both MaNAG1 and MaNAG2 have predicted secretion signal peptides, from which extracellular functions can be inferred. In contrast, putative GH3 NAGases are apparently cytoplasmic enzymes as they do not present any predicted secretion signals.

### Phylogeny of putative GH20 NAGases

Twenty-six MaNAG1 and MaNAG2 orthologs were identified in 15 filamentous fungi genomes. Most of them are single copy of each putative GH20 NAGase of *M. anisopliae*. The conserved motif of GH20 proteins and the highly conserved catalytic residues, aspartic and glutamic acids (D-E), were recognized in all of GH20 orthologs (Figure S1).

**Figure 3:**
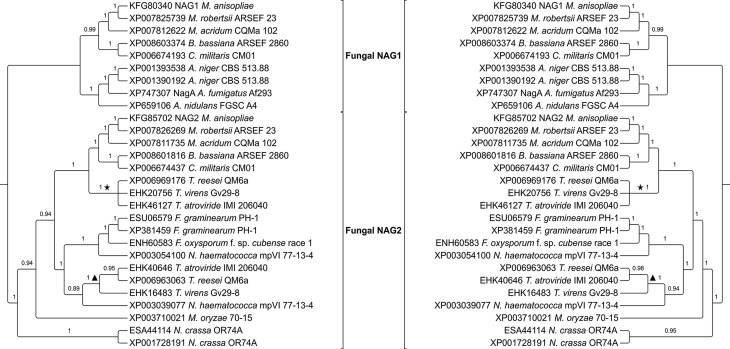
Phylogenetic relationships among GH20 NAGases from filamentous fungi. The phylogenies were obtained using MrBayes 3.2.5 (left side) and PhyML 3.1 (right side). ▲: *Trichoderma* NAG1. ★: *Trichoderma* NAG2.

The best-fit evolutionary model for GH20 NAGases was LG+I+G, which was used for the phylogenetic inference. Phylogenetic analyses of GH20 NAGases from *M. anisopliae* and the other fifteen ascomycetes revealed an early duplication event in GH20 NAGases, resulting in two distinct main clades (Figure 3). MaNAG1 formed a monophyletic group with other entomopathogenic fungi NAGase sequences (*Metarhizium robertsii*, *Metarhizium acridum*, *Cordyceps militaris* and *Beauveria bassiana*). This cluster also formed a statistically supported clade with species from the *Aspergillus* genus. In contrast, MaNAG2 exhibits a more diverse evolutionary history, with orthologs present in *Trichoderma* sp*.*, *Fusarium* sp, *Neurospora* sp., and *Magnaporthe* sp. Interestingly, the present evolutionary analysis revealed that both NAG1 and NAG2 from the mycoparasite *T. atroviride*, used in the *M. anisopliae* genome screening, are evolutionarily more related to MaNAG2 (Figure 3).

For the majority of the 15 fungi analyzed, only one ortholog to MaNAG1 and one ortholog to MaNAG2 were detected in each species. Duplication events on a specific lineage resulting in paralogous proteins was only observed for *Aspergillus niger*, which has two MaNAG1 orthologs, and for *Nectria haematococca*, *N. crassa* and *Fusarium graminearum*, with two MaNAG2 orthologs.

### Phylogeny of putative GH3 NAGases

Twenty-three MaNAG3 and MaNAG4 orthologs were identified on the filamentous fungi genomes examined. Conserved sequence motifs of GH3 proteins (K-H-F / I-P-G-H / L-G-x-x-x-x-D-S / T-H) were found in all of them, however, few amino acid residues substitutions were observed (Figure S2). All 15 filamentous fungi have MaNAG3 orthologs. However, the *M. acridum* gene ortholog was not included in the phylogenetic analysis, since it was not properly annotated in the *M. acridum* genome. In turn, only *Trichoderma* sp., *Aspergillus* sp., and the entomopathogens *C. militaris* and *B. bassiana* have MaNAG4 orthologs.

To better understand GH3 NAGases evolutionary relationships, 10 well described bacterial GH3 NAGases and the characterized GH3 NAGase from the zygomycete *R. Miehei* (Yang *et al.*, 2014) were added to the phylogenetic analysis (Table 1). Since several GH3 family fungal members with β-glucosidase activity have also been described (Kawaguchi *et al.*, 1996; Krogh *et al.*, 2010; Nakajima *et al.*, 2012; Kudo *et al.*, 2015; Zhao *et al.*, 2015; Colabardini *et al.*, 2016; Pei *et al.*, 2016), the phylogenetic relationships among the fungal, bacterial, and *R. miehei* GH3 NAGases were inferred including as outgroup putative β-glucosidases from *M. anisopliae* E6, characterized fungal β-glucosidases and putative β-glucosidases from species described in Table 1. The best-fit evolutionary model for GH3 NAGases was LG+I+G. The evolutionary relationship of all GH3 proteins showed two distinct clades separating fungal and bacterial NAGases from β-glucosidases (Figure 2).

The phylogenetic tree revealed that MaNAG3 and MaNAG4 orthologs formed two distinct clusters (Figure 2). Both MaNAG3 and MaNAG4 grouped to other *Metarhizium* species, but in contrast with the GH20 NAGases phylogeny, putative GH3 NAGases from *Metarhizium* sp. are evolutionarily more distant from putative GH3 NAGases of other entomopathogenic fungi (*B. bassiana* and *C. militaris*). Additionally, gene duplication of MaNAG3 and MaNAG4 orthologs was not observed.

Bacterial sequences did not form a monophyletic group, but they are basal in relation to fungal NAG3 and NAG4 (Figure 2). The difference between bacterial NAGases apparently is not related to gram-positive or gram-negative structural classification. It was also observed that even bacterial NAGases with high chitinolytic substrate specificity (*S. thermoviolaceus* NagA, *Clostridium paraputrificum* NagZ, *Alteronomonas sp.* HexA, *V. furnissii* NagZ, *Thermotoga maritma* NagA and *T. neapolitana* CbsA) grouped into distinct clades from fungal NAGases. This is probably due to the fact that some bacterial NAGases do not necessarily have GlcNAc hydrolysis specificity over chitooligosaccharides. For example, *E. coli* NagZ cleaves GlcNAc from muropeptides present in the bacterial cell wall (Cheng *et al.*, 2000). *C. fimi* Nag3 is also an unusual GH3 NAGase, because it is a β-N-acetylhexosaminidase with a wide range of substrates, hydrolyzing both β-N-acetylglucosaminedes and β-glucosides (Mayer *et al.*, 2006).

### Patterns of transcript relative expression of putative NAGases

The expression profile of *M. anisopliae* putative NAGases was investigated in different cell types under different culture conditions: mycelium grown on glucose 1%, GlcNAc 0.25%, chitin 1% or autolysis conditions; and induced conidia, blastospore and appressorium. The four putative NAGase gene transcripts were detected in all *M. anisopliae* cell types and culture conditions, validating the annotation of the proposed genes.

**Figure 4:**
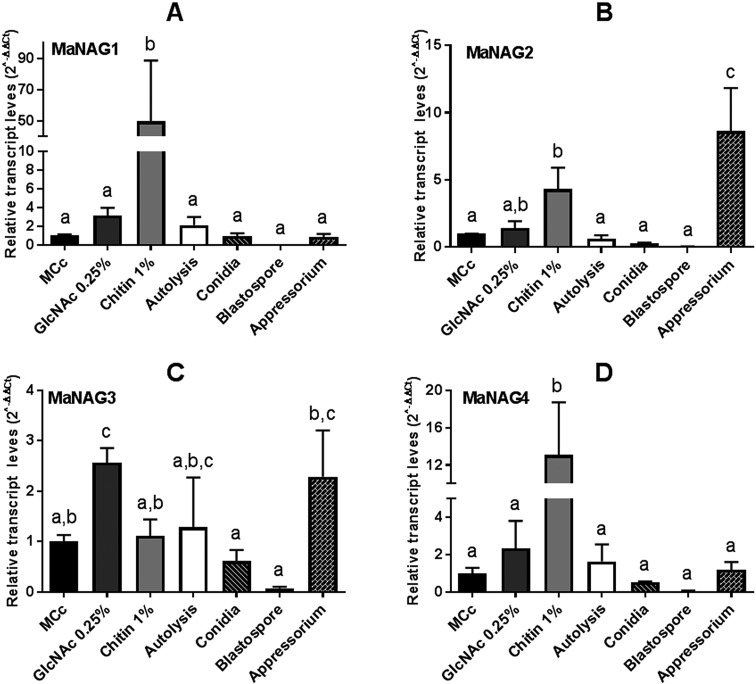
Relative expression of GH20 and GH3 NAGase genes in *M. anisopliae*, considering MCc as the reference condition. Transcriptional profiles of GH20 NAGase genes (MaNAG1 and MaNAG2) and GH3 NAGase genes (MaNAG3 and MaNAG4) in seven different conditions (mycelium growth on different carbon source media, autolysis, and different cell types), using *tef1*α as a reference gene and applying the 2^-△△Ct^ method. A) *nag1*; B) *nag2*; C) *nag3*; D) *nag4*. Standard error bars are indicated. Different letters indicate statistically significant differences (*p* < 0.05) among studied conditions.

To gain information on the regulation of the putative NAGases by substrate, the transcript level of genes from *M. anisopliae* cultured in MCc medium was established as a reference condition (Figure 4). Interestingly, the expression of MaNAG1, MaNAG2 and MaNAG4 were induced by 1% chitin, albeit at different levels (Figure 4). Notably, MaNAG1 showed the most pronounced expression induction on this carbon source (Figure 4A). Additionally, MaNAG3 was the only MaNAGase induced in cultures with added 0.25% GlcNAc (Figure 4C). When different cellular types were taken into account, MaNAG3 exhibited detectable transcripts in cells forming appressorium, while MaNAG2 was strongly induced in this cell type (Figure 4B). The expression of the four putative NAGases gene showed only basal levels in conidia and blastospores (Figure 4). These results indicate the minor participation of putative GH3 and GH20 in conidia and blastospores.

## Discussion

Virulence determinants are the main focus in the study of entomopathogenic fungi (Schrank and Vainstein, 2010). As chitin is present in the exoskeleton of several arthropods, enzymes involved in chitin degradation and assimilation are predicted to play essential roles in host-entomopathogen interactions (Schrank and Vainstein, 2010). While chitinases are widely explored in entomopathogens and several fungal species with diverse pathogenic traits, the role of NAGases in the fungal life cycle and their importance in infection has not been explored. Here, four putative NAGase genes belonging to the GH20 family (*MaNAG1* and *MaNAG2*) and GH3 family (*MaNAG3* and *MaNAG4*) of *M. anisopliae* genome were analyzed.

St. Leger *et al.* (1991) purified a secreted NAGase from *M. anisopliae* by gel-filtration, with a pI of 6.4 and molecular mass of 110-120 kDa. We hypothesize that this *M. anisopliae* purified enzyme could be the MaNAG1 presented here, based on the predicted pI (6.07) and molecular mass (66.98 kDa) of MaNAG1, likely forming a homodimer. In fact, some fungal GH20 NAGases (Koga *et al.*, 1991; Rylavá *et al.*, 2011) and some bacterial GH3 NAGases (Choi *et al.*, 2009) exhibit a homodimer composition. Nevertheless, the molecular characterization of *M. anisopliae* putative NAGases will be necessary to determinate if the dimer structure is relevant to enzymatic activity.

Phylogenetic analyses of putative GH20 NAGases revealed the occurrence of at least one duplication event before its divergence in fungi. This early gene duplication is supported by evolutionary analysis of GH20 family from several eukaryotic taxa, reported by Intra *et al.* (2008). Comparing the evolutionary history of MaNAG1 and MaNAG2, subsequent duplication events resulted in current presence of multiple GH20 NAGase orthologs in ascomycetes. This phenomenon was more frequent in the MaNAG2 than the MaNAG1 cluster, culminating in the presence of MaNAG2 orthologs in a broader spectrum of fungi with different lifestyles. While MaNAG1 has orthologs only in entomopathogens and in the saprophytic/human pathogens *Aspergillus* sp*.*, MaNAG2 orthologs are present in entomopathogens, mycopathogen species, such as *Trichoderma* sp., phytopathogens including *N. haematococca*, *Fusarium* sp*.* and *M. oryzae*, and in saprophytes, such as *N. crassa*. These species belong to distinct orders, however, a previous study has observed their close evolutionary relationship (Wang *et al.*, 2009). The widespread presence of MaNAG2 orthologs in fungi with diverse lifestyles could represent a common basic function for all these enzymes despite differences in fungal lifestyles, while MaNAG1 would have more specific roles in an entomopathogenic lifestyle.

In our analysis, *M. anisopliae* and *M. robertsii* formed a statistically well supported clade, with *M. acridum* as a basal species, corroborating the phylogeny relationships among these *Metarhizium* species (Bischoff *et al.*, 2009; Staats *et al.*, 2014). Our results revealed a close evolutionary relationship of GH20 NAGases between the *Metarhizium* clade and the one formed by *Beauveria* and *Cordyceps* genera. The conservation of secreted proteins in fungi has been observed among *M. anisopliae* and entomopathogens *Metarhizium* spp*.*, *B. bassiana* and *C. militaris* (Staats *et al.*, 2014). Therefore, the evolutionary pattern of GH20 NAGases in entomopathogens is representative of the extremely similar evolutionary pattern of all secreted proteins found in fungi with similar hosts.

The glycoside hydrolases from the CAZy family GH3 display an unusual diversity in structure, specificity, and biological roles (Macdonald *et al.*, 2015). In many cases the enzymes have dual or broad substrate specificities with respect to monosaccharide residues, linkage position and chain length of the substrate. This family harbors members with several activities, most notably β-glucosidases and NAGases (Macdonald *et al.*, 2015). Several fungal β-glucosidases from the GH3 family have been characterized (Kawaguchi *et al.*, 1996; Krogh *et al.*, 2010; Nakajima *et al.*, 2012; Kudo *et al.*, 2015; Zhao *et al.*, 2015; Colabardini *et al.*, 2016; Pei *et al.*, 2016), however the first fungal NAGase from family GH3 was only recently described (RmNag) (Yang *et al.*, 2014).

The first goal of our phylogenetic analyses was to clearly distinguish putative NAGases from putative β-glucosidases. The phylogenetic analysis set apart putative GH3 NAGases from putative GH3 β-glucosidases (Figure 3), suggesting GH3 NAGase activity for MaNAG3 and MaNAG4. Indeed, the characterized RmNag clustered together with MaNAG3 and MaNAG4 with robust support, suggesting the possibility of similar functions of these enzymes (Figure 3). The five other putative GH3 proteins from *M. anisopliae* (KFG84234, KFG86760, KFG85258, KFG81708, and KFG84481) clustered together with characterized β-glucosidases, again suggesting the possibility of similar function (Figure 2). Additionally, several characterized bacterial GH3 NAGases were more phylogenetically related with MaNAG3, MaNAG4 and RmNag than with the characterized β-glucosidases (Figure 2).

Bacterial NAGases were added to phylogenetic analyses, which show well-established acetyl-chitooligosaccharide degradation activity, and NAGases with other substrate specificities, such as NagZ from *E. coli* and NAG3 from *C. fimi*. *E. coli* NagZ participates in bacteria cell wall recycling by hydrolyzing GlcNAc from muropeptides (Cheng *et al.*, 2000). In turn, *C. fimi* NAG3 was identified as a bifunctional β-N-acetyl-D-glucosaminidase/β-D-glucosidase (Mayer *et al.*, 2006). It was also reported that *C. fimi* NAG3 enzyme is actually a GlcNAc-phosphorylase using phosphate rather than water as nucleophile (Macdonald *et al.*, 2015). Macdonald’s study suggests that other GH3 NAGases can harbor GlcNAc-phosphorylase activity. Notably, our GH3 phylogenetic analysis showed that *C. fimi* NAG3 has a basal position in relation to other bacterial and fungal NAGases with chitin specificity, supporting this suggestion. However, complementary experiments are required to evaluate this putative GlcNAc-phosphorylase activity.

The phylogenetic analysis of putative GH3 NAGases suggests an early acquisition of GH3 NAGases in fungi, indicating that the observed diversity resulted from ancient duplications that occurred after the divergence between bacteria and the fungi GH3 family genes (Figure 3). Fungal orthologs of MaNAG3 and MaNAG4 formed two distinct clades. In relation to the NAG4 clade, MaNAG4 was arranged closer to GH3 NAGases of the mycoparasitic *Trichoderma* sp. than orthologs of entomopathogenic species *C. militaris* and *B. bassiana.* The NAG3 from entomopathogens formed a monophyletic group with *Trichoderma* species, with MaNAG3 basal to them. It seems that *M. anisopliae* GH3 NAGases may not have specific roles in entomopathogenic fungal lifestyle. However, at this point, their participation in basal cell processes cannot be ruled out, such as GlcNAc carbon metabolism and cell wall remodeling, both processes necessary to hyphal growth and cell differentiation.

The qPCR assays of putative GH20 and GH3 NAGase genes confirmed that the identified sequences are functional. *M. anisopliae* putative NAGases showed differential transcript profiles in response to different conditions, indicating an absence of a common gene regulation pattern. These variable expression profiles also suggest they may not have totally redundant roles. *M. anisopliae* GH20 NAGases, *MaNAG1* and *MaNAG2*, exhibited induced expression patterns when cultured in the presence of 1% chitin. Our results reflect the well-established condition, where chitin induces the expression of secreted chitinolytic enzymes (St. Leger *et al.*, 1991; Seidl, 2008). The presence of a predicted signal peptide for secretion in MaNAG1 and MaNAG2, and their expression induction by chitin reveal their probable role in extracellular chitinolytic activity in *M. anisopliae,* acting on extracellular cleavage of chitobiose into GlcNAc monomers for the assimilation of this carbon source. Nonetheless, it is important to note that other carbon sources are also able to stimulate, at lower levels, the expression of GH20 NAGases (Seidl *et al.*, 2006).

The expression profile of *M. anisopliae* putative NAGases in appressorium is noteworthy. MaNAG2 was the most significantly expressed NAGase in appressorium, being highly induced in this cell type. The appressorium is a specialized penetration structure that helps to dissolve the host chitinous exoskeleton. These cells use enzyme secretion and physical pressure to mediate penetration. Therefore, it can be suggested that MaNAG2 is putatively required at early stages of infection, during the penetration stage or to remodel the fungal cell wall in appressorium differentiation.

It was expected that 0.25% GlcNAc would induce *M. anisopliae* NAGases, because this low concentration of GlcNAc is described as an inducer for chitinolytic genes, and only high monomer concentrations (> 0.5%) would repress expression of chitinolytic enzymes by their own activity products in *M. anisopliae* (catabolic repression) (Barreto *et al.*, 2004). Similarly, *T. atroviride nag1* expression is induced by GlcNAc (Mach *et al.*, 1999). However, the transcript expression analysis showed that *M. anisopliae* putative GH20 NAGases were not induced by GlcNAc. MaNAG3 was the only putative NAGase induced by 0.25% GlcNAc and the only putative NAGase that was not induced by 1% chitin, suggesting a possible regulatory mechanism for this gene, in which the expression could depend on the prior degradation of chitin to GlcNAc. Furthermore, no transcript induction of any NAGases was observed in blastospores and conidia (Figure 4), which are cellular forms with diminished metabolic activity, although not completely dormant (Novodvorska *et al.*, 2016). In addition, *M. anisopliae* conidial extracts and immunoproteomic analysis indicate that chitinases may be localized on the conidial surface (Santi *et al.*, 2009, 2010), NAGase activity is probably not necessary in these resting cells. In contrast, blastospores are cell types that facilitate dispersal in host hemolymph during colonization. At this stage, the fungus has already transposed the chitinous exoskeleton and uses trehalose and other carbon sources, not requiring, necessarily, the expression of chitinolytic enzymes (Xia *et al.*, 2002). Nevertheless, it is important to note that the ADAMEK media used to induce blastospores does not fully mimic the arthropod inner body complexity, and GH3 and GH20 NAGase activity may be required in specific steps of blastospore differentiation and infection.

Chitinases and NAGases act consecutively and synergistically to render complete degradation of chitin. This may be the result of common regulation patterns between these two groups of enzymes, as revealed in *T. atroviride* NAGase studies (Tharanathan and Kittur, 2003). The experimental conditions used in this study for the evaluation of *M. anisopliae* putative NAGase expression were the same employed for the study of the 21 chitinases from *M. anisopliae* (Junges *et al.*, 2014). This allowed the comparison of the performance of different genes of the chitinolytic process to propose potential relationships between specific chitinases and NAGases. Junges *et al.* (2014) described a large group of chitinases induced by chitin: *chimaA1*, *chimaA6*, *chimaA8*, *chimaB1*, *chimaB2*, *chimaB3*, *chimaB4*, *chimaB6*, *chimaC3*. This expression pattern can be associated with *MaNAG1*, *MaNAG2* and *MaNAG4* that also displayed increased expression profile in the presence of chitin. Also, those putative NAGases induced by chitin could be followed by chitinase action induced by GlcNAc monomers (*chimaD1*) (Junges *et al.*, 2014). On media supplemented with the GlcNAc monomer, the MaNAG3 gene showed strong expression when compared to the other *M. anisopliae* putative NAGases, coinciding with the *chimaD1* chitinase pattern. Moreover, in the induced apressorium formation condition, the expression of MaNAG2 could be related to *chimaA5* chitinase, since both are overexpressed in this cellular type.

Our results are in agreement to previous suggestions of the presence of GH3 NAGases in fungi (RmNag) (Yang *et al.*, 2014) and in the Hypocreales order (Kappel *et al.*, 2016). In fact, Kappel *et al.* (2016) have functionally characterized a GH3 gene (named *nag3*; XP006966911) in *T. reesei,* the product of which, an MaNAG3 ortholog, holds suggested NAGase activity. The phylogenetic analyses indicate that MaNAG3 and *T. reesei* NAG3 are phylogenetically related (Figure 2). The existence of more putative NAGase genes argues that the genomic arsenal of NAGases in ascomycetes is not as small as previously thought, attenuating the discrepancy between the number of chitinase and NAGase genes. It is also not possible to rule out the existence of other unknown NAGases in *M. anisopliae* and other fungal species. In this sense, we have identified a fifth and unexplored putative NAGase gene in *M. anisopliae*, belonging to the GH84 family. The product of this gene (KFG85933.1) exhibits 63% identity with characterized GH84 from *Penicillium chrysogenum* (XP_002557703.1). The *P. chrysogenum* GH84 NAGase not only exhibit activity against GlcNAc substrates, but also hydrolyzes substrates with *galacto*-configuration and exhibits transglycosylation activity (Slámová *et al.*, 2014).

In conclusion, this study explored relevant evolutionary aspects of putative GH3 and GH20 NAGase genes and the expression analysis highlighted possible functions for these genes in *M. anisopliae* and entomopathogenic fungi. This analysis will allow the selection of genes for further functional characterization to elucidate the process and to identify redundancies and specificities. The view that chitinase diversity is merely redundant may not correct (Seidl *et al.*, 2005; Tzelepis *et al.*, 2012; Junges *et al.*, 2014). However, the strategy of constructing deleted strains is not always straightforward to determine function (Alcazar-Fuoli *et al.*, 2011). Here, *M. anispoliae* putative GH20 NAGase genes revealed induced transcript production in the presence of chitin, potentially in the extracellular milieu. The detection of *MaNAG3* and *MaNAG4* putative genes is the first evidence for the presence of a possible GH3 family of NAGases in entomopathogenic fungi. *MaNAG3* and *MaNAG4* expression is responsive to chitinous substrates, suggesting their potential influence on cell differentiation during the *M. anisopliae* life cycle.

## Supplementary material

The following online material is available for this article:

Table S1 - Primer sequences.

Figure S1 - Major *Metarhizium anisopliae* cell types involved in the cycle of infection.

Figure S2 - Multiple alignment of GH20 NAGases from filamentous fungi.

Figure S3 - MaNAG4 nucleotide and amino acid sequence.

Figure S4 - Multiple alignment of GH3 NAGases from bacteria, zygomycetes, filamentous fungi, and *M. anisopliae* β-glucosidases.
