# Erratum: Molecular evolution and transcriptional profile of GH3 and GH20 ß-N-acetylglucosaminidases in the entomopathogenic fungus Metarhizium anisopliae

**DOI:** 10.1590/1678-4685-GMB-2017-0363er

**Published:** 2019-02-28

In the article "Molecular evolution and transcriptional profile of GH3 and GH20 ß-N-acetylglucosaminidases in the entomopathogenic fungus *Metarhizium anisopliae*", with DOI number 10.1590/1678-4685-gmb-2017-0363, published in the journal Genetics and Molecular Biology, 41(4):843-857, on page 843, a footnote informing the current address of the following author was omitted: Claudia Elizabeth Thompson, Departamento de Farmacociências, Universidade Federal de Ciências da Saúde de Porto Alegre, Rua Sarmento Leite, 245, 90050-170 Porto Alegre, RS, Brazil.

