# Supplementary material for: Molecular evolution and transcriptional profile of GH3 and GH20 β-N-acetylglucosaminidases in the entomopathogenic fungus Metarhizium anisopliae
Source: Genet Mol Biol. 2018 Dec 10;41(4):843–57. doi: 10.1590/1678-4685-GMB-2017-0363 (PMC6415606; doi:10.1590/1678-4685-GMB-2017-0363)
Supplement: Supplementary file 1 [file 1415-4757-GMB-1678-4685-GMB-2017-0363-s002.pdf]

Supplementary Material to "Molecular evolution and transcriptional profile of GH3 and GH20 β-N-acetylglucosaminidases in the entomopathogenic fungus Metarhizium anisopliae"

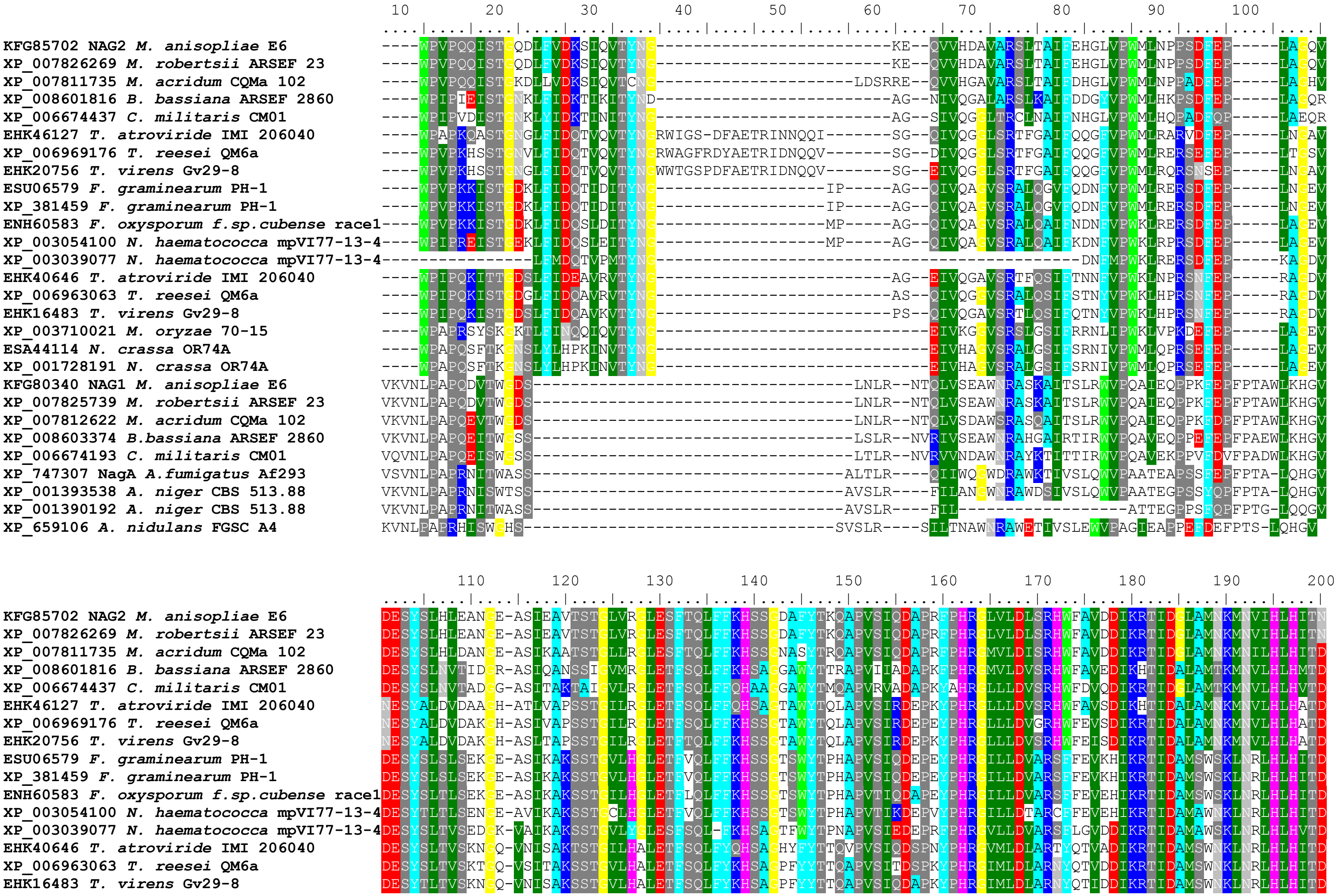

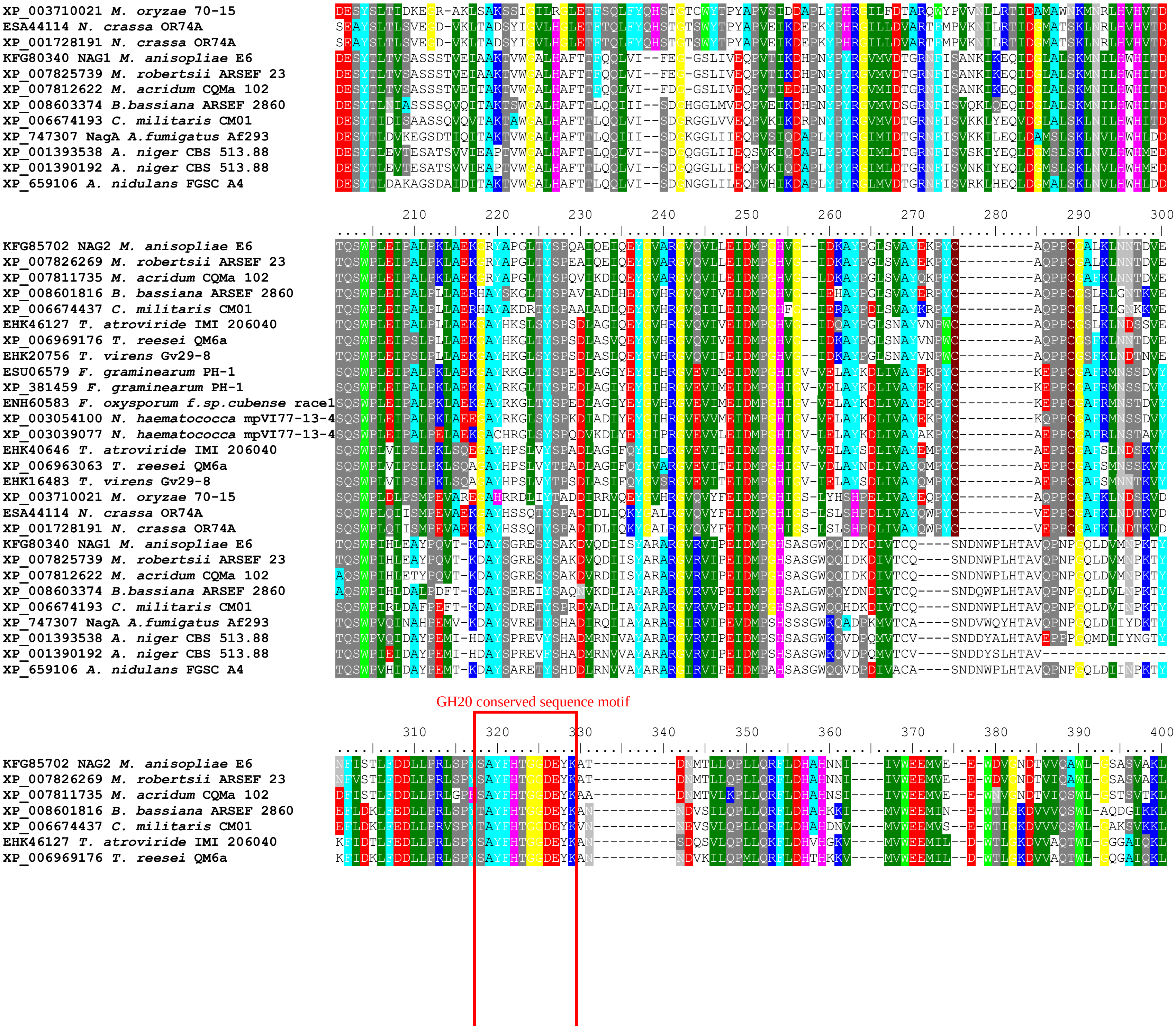
GH20 conserved sequence motif
XP_003710021 M. oryzae 70-15
ESA44114 N. crassa OR74A
XP_001728191 N. crassa OR74A
KFG80340 NAG1 M. anisopliae E6
XP_007825739 M. robertsii ARSEF 23
XP_007812622 M. acridum CQMa 102
XP_008603374 B.bassiana ARSEF 2860
XP_006674193 C. militaris CM01
XP_747307 NagA A.fumigatus Af293
XP_001393538 A. niger CBS 513.88
XP_001390192 A. niger CBS 513.88
XP_659106 A. nidulans FGSC A4
KFG85702 NAG2 M. anisopliae E6
XP_007826269 M. robertsii ARSEF 23
XP_007811735 M. acridum CQMa 102
XP_008601816 B. bassiana ARSEF 2860
XP_006674437 C. militaris CM01
EHK46127 T. atroviride IMI 206040
XP_006969176 T. reesei QM6a
EHK20756 T. virens Gv29-8
ESU06579 F. graminearum PH-1
XP_381459 F. graminearum PH-1
ENH60583 F. oxysporum f.sp.cubense race1
XP_003054100 N. haematococca mpVI77-13-4
XP_003039077 N. haematococca mpVI77-13-4
EHK40646 T. atroviride IMI 206040
XP_006963063 T. reesei QM6a
EHK16483 T. virens Gv29-8

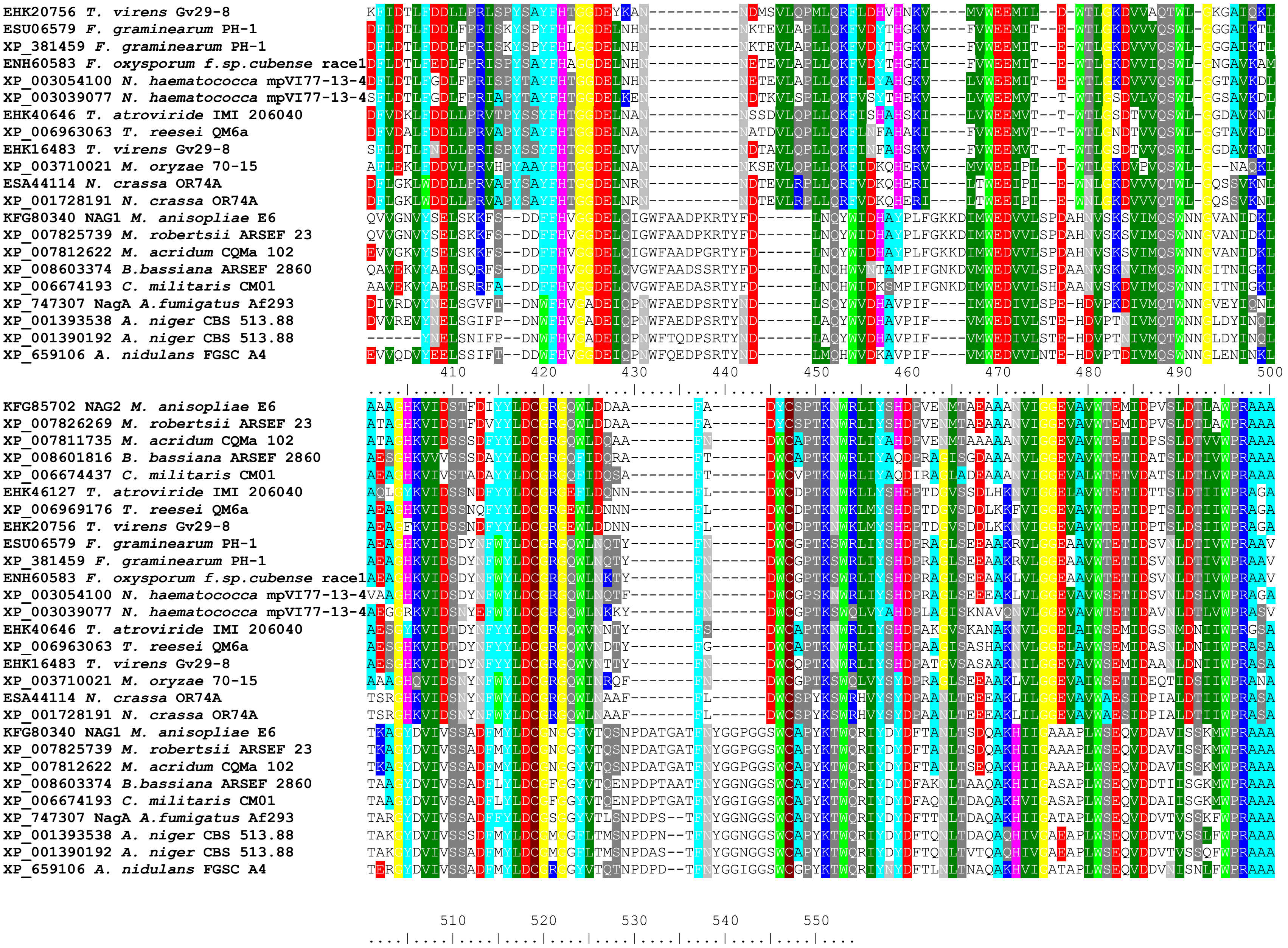
EHK20756 T. virens Gv29-8
ESU06579 F. graminearum PH-1
XP_381459 F. graminearum PH-1
ENH60583 F. oxysporum f.sp.cubense race1
XP_003054100 N. haematococca mpVI77-13-4
XP_003039077 N. haematococca mpVI77-13-4
EHK40646 T. atroviride IMI 206040
XP_006963063 T. reesei QM6a
EHK16483 T. virens Gv29-8
XP_003710021 M. oryzae 70-15
ESA44114 N. crassa OR74A
XP_001728191 N. crassa OR74A
KFG80340 NAG1 M. anisopliae E6
XP_007825739 M. robertsii ARSEF 23
XP_007812622 M. acridum CQMa 102
XP_008603374 B.bassiana ARSEF 2860
XP_006674193 C. militaris CM01
XP_747307 NagA A.fumigatus Af293
XP_001393538 A. niger CBS 513.88
XP_001390192 A. niger CBS 513.88
XP_659106 A. nidulans FGSC A4
410 420 430 440 450 460 470 480 490 500
KFG85702 NAG2 M. anisopliae E6
XP_007826269 M. robertsii ARSEF 23
XP_007811735 M. acridum CQMa 102
XP_008601816 B. bassiana ARSEF 2860
XP_006674437 C. militaris CM01
EHK46127 T. atroviride IMI 206040
XP_006969176 T. reesei QM6a
EHK20756 T. virens Gv29-8
ESU06579 F. graminearum PH-1
XP_381459 F. graminearum PH-1
ENH60583 F. oxysporum f.sp.cubense race1
XP_003054100 N. haematococca mpVI77-13-4
XP_003039077 N. haematococca mpVI77-13-4
EHK40646 T. atroviride IMI 206040
XP_006963063 T. reesei QM6a
EHK16483 T. virens Gv29-8
XP_003710021 M. oryzae 70-15
ESA44114 N. crassa OR74A
XP_001728191 N. crassa OR74A
KFG80340 NAG1 M. anisopliae E6
XP_007825739 M. robertsii ARSEF 23
XP_007812622 M. acridum CQMa 102
XP_008603374 B.bassiana ARSEF 2860
XP_006674193 C. militaris CM01
XP_747307 NagA A.fumigatus Af293
XP_001393538 A. niger CBS 513.88
XP_001390192 A. niger CBS 513.88
XP_659106 A. nidulans FGSC A4
510 520 530 540 550

```
KFG85702 NAG2 M. anisopliae E6                AGEAWWSGRKDG-EGNLRSVFTARPRLEEMRERMLARGVRGAVISQLFCGQ---
XP_007826269 M. robertsii ARSEF 23           AGEAWWSGRRDG-EGNLRSVFTARPRLEEMRERMLARGVRGAVISQLFCGQ---
XP_007811735 M. acridum CQMa 102             AGEAWWSGRRDG-EGNLRSVYTARPRLGEMRERMLVRGVRGAVISQLFCGQ---
XP_008601816 B. bassiana ARSEF 2860          AGESLWSSRYES-DGKNRSMYDVRPRLSEMRERMLARGVRGAPITQLWCDQ-E-
XP_006674437 C. militaris CM01               AGESWWSGRSGA-DGKNRSMYEVRPRMSEMRERMLARGVRGAPITQLWCDQ-E-
EHK46127 T. atroviride IMI 206040            AAEVWWSGRVDEATGTNRSQLDARPRLSEQRERMLARGVRGAPITQLWCSQ--G
XP_006969176 T. reesei QM6a                  AAEIWWSGKTDE-HGANRSQIDARPRLSEQRERMLARGVRGTPITQLWCSQ--G
EHK20756 T. virens Gv29-8                    AAEIWWSGKIDE-KGNNRSQIDARPRLSEQRERMLARGVRGTPITQLWCSQ--G
ESU06579 F. graminearum PH-1                 MGEVLWSGRTDAS-GQNRSQYDAAPRLAEMRERMVARGVSASPVQMPFCTQ---
XP_381459 F. graminearum PH-1                MGEVLWSGRTDAS-GQNRSQYDAAPRLAEMRERMVARG----------------
ENH60583 F. oxysporum f.sp.cubense race1     MGEVLWSGRTDAS-GQNRSQYDAAPRLAELRERMVARGVSASPIQMPFCTQ---
XP_003054100 N. haematococca mpVI77-13-4     AGEVLWSGRTDAS-GKNRTQYDAAPRLAEMRERMVARG----------------
XP_003039077 N. haematococca mpVI77-13-4     AGEVLWSGRQDAA-GQNRSQYDAMPRLAEFRERLVARGLRTSPIQMTFCTQ---
EHK40646 T. atroviride IMI 206040            AGEVWWSGNVDTATGQNRSQLEVTPRLNEFRERMLARGVNAMPIQMTYCTQ---
XP_006963063 T. reesei QM6a                  AGEVWWSGNVDAATGQNRSQLEVVPRLNEFRERMLARGVSAMPIQMTYCTQ---
EHK16483 T. virens Gv29-8                    AGEVWWSGNADPATGQQRSQLDVVPRLNEFRERLLARGVSAMPIQMTYCTQ---
XP_003710021 M. oryzae 70-15                 AGEVLWSGRIDPATGQNRSQLEAIPRLSEMRERLVARGVRPAALTQLWCTQ---
ESA44114 N. crassa OR74A                     AGEVLWSGRIDPATGQNRTQLDAAPRLSELRERLVARGVQSSSVYMTWCTQD--
XP_001728191 N. crassa OR74A                 AGEVLWSGRIDPATGQNRTQLDAAPRLSELRERLVARGVQSSSVYMTWCTQD--
KFG80340 NAG1 M. anisopliae E6                LAELVWSGNKDPKTGLKRTTY-LTQRILNFREYLVANGIGAAPLVPKYCLQHTA
XP_007825739 M. robertsii ARSEF 23           LAELVWSGNKDPKTGLKRTTY-LTQRILNFREYLVANGIGAAPLVPKYCLQHTA
XP_007812622 M. acridum CQMa 102             LAELVWSGNKDPKTGLKRTTY-LTQRILNFREYLVANGIGAAPLAPKYCLQHTA
XP_008603374 B.bassiana ARSEF 2860           LGELVWSGNRDPKTGKKRTTS-FTQRILNFREYLVANGIGATALVPKYCLQHDA
XP_006674193 C. militaris CM01               LAELLWSGNRDPKTGKKRTTT-FTQRILNFREYLVANGVAATPLVPKYCLQHEA
XP_747307 NagA A.fumigatus Af293            LAELVWSGNRDAN-GKKRTTL-MTQRILNFREYLLANGIQAGNLVPKYCLQHSA
XP_001393538 A. niger CBS 513.88           LAELVWSGNRDEN-GHKRTTL-MTQRILNFREYLVANGVQAKALVPKYCVQRSV
XP_001390192 A. niger CBS 513.88           LAELVWSGNRDEN-GRKRTTL-MTQRILNFREYLVANGAQAQALVPKYCVQHSA
XP_659106 A. nidulans FGSC A4              LAELVWSGNRDAK-GNKRTTL-FTQRILNFREYLLANGVMAATVVPKYCLQHTV
```

Figure S2 - Multiple alignment of GH20 NAGases from filamentous fungi. Amino acid alignment built and trimmed with GUIDANCE2 using PRANK as the MSA algorithm with 100 bootstrap replicates. Location of the GH20 conserved sequence motif (H/N-x-G-A/C/G/M-D-E-A/I/L/V) is highlighted.
