# Supplementary material for: Molecular evolution and transcriptional profile of GH3 and GH20 β-N-acetylglucosaminidases in the entomopathogenic fungus Metarhizium anisopliae
Source: Genet Mol Biol. 2018 Dec 10;41(4):843–57. doi: 10.1590/1678-4685-GMB-2017-0363 (PMC6415606; doi:10.1590/1678-4685-GMB-2017-0363)
Supplement: Supplementary file 2 [file 1415-4757-GMB-1678-4685-GMB-2017-0363-s001.pdf]

Supplementary Material to "Molecular evolution and transcriptional profile of GH3 and GH20 β-N-acetylglucosaminidases in the entomopathogenic fungus Metarhizium anisopliae"

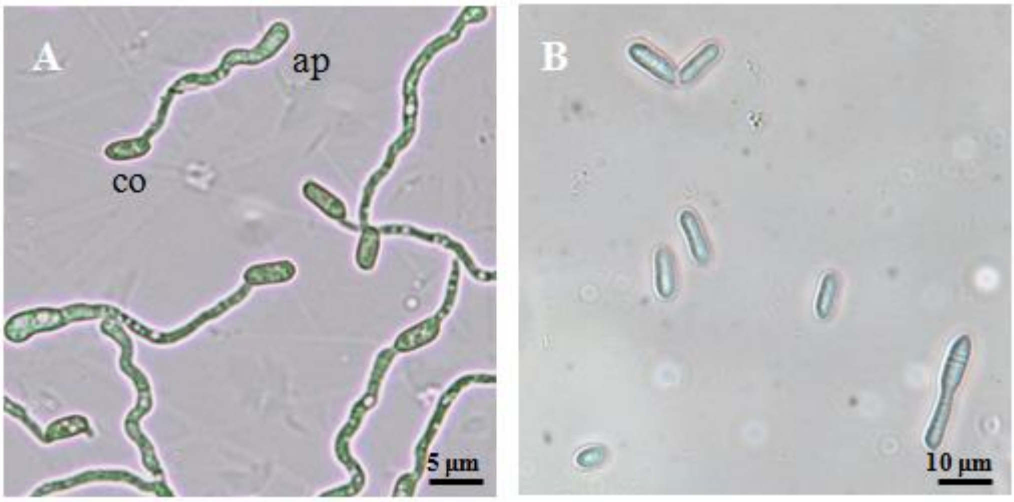

Figure S1 - Major *Metarhizium anisopliae* cell types involved in the cycle of infection and analyzed in this study. (A) A germinating conidium producing an appressorium, induced over glass coverslips; (B) Budding yeast-type cells (blastospores) produced by the fungus to facilitate dispersal in insect hemocoel. co: conidium; ap: appressorium.
