# Supplementary material for: Molecular evolution and transcriptional profile of GH3 and GH20 β-N-acetylglucosaminidases in the entomopathogenic fungus Metarhizium anisopliae
Source: Genet Mol Biol. 2018 Dec 10;41(4):843–57. doi: 10.1590/1678-4685-GMB-2017-0363 (PMC6415606; doi:10.1590/1678-4685-GMB-2017-0363)
Supplement: Supplementary file 3 [file 1415-4757-GMB-1678-4685-GMB-2017-0363-s003.pdf]

Supplementary Material to "Molecular evolution and transcriptional profile of GH3 and GH20 β-N-acetylglucosaminidases in the entomopathogenic fungus Metarhizium anisopliae"

MaNAG4 nucleotide sequence

ATGAGACCCTCTATCATTTGCGACCGAGACGCCTCTCGACCCCTCACTGGAGGTGTCCAACCGTCA
ACTCGGAACATCAAACCAGCTCCAACCCTCATCTTCCCCACGGACCCGGCATGCATGGGGCTCTTT
GCGGTTGGCTTCTACGGGACCGAAATCAACAAAGAAATCAAGTCTCTCATCCAAGACTATGGCGTG
GGCGCCGTTCTCCTCTTCAAGCGCAACATCAAAGACGCCGCCCAGCTACAAGCTCTCTGCCTGGGA
CTTCAACAACTGGCCCAGGATGCCGGCCACACCCAGCCTCTCTTTGTCGGCATCGACCAGGAAAAC
GGACTCGTGACGCGCATTTCACCTCCAGTCGCGGCACAGCTGCCCGGACCAATGGCCCTGGGCGCC
GCCGCATCGTTGGAGAGCGCATACCACGTCGCCAAAGCCACGGGCAACATGTTGCGCTATTTCGGC
ATCAACATGAATTACGCCCCAGTCGGCGACGTCAATAACGAGCCGCTGAACCCCGTCATTGGCGTA
CGGAGTCCCGGCGACGACGCCGACAAGGTCGCCCGATTTGCCGCCGAGTGTGCCAAGGGCCTGCGA
GAGACCAGAGTCGCGCCCTGCATCAAGCACTTCCCGGGCCACGGGGACACGGCCGTAGATTCGCAC
TACGGCCTGCCCGTCGTCAACAAGACCAGGGGGGAGCTGGAAGCGCTGGAGCTGGTGCCCTTTCGC
CGCGCGGCCGCAGAGGGCATCGAGATGGTCATGACGGCGCACATTGCCCTCCCCAAGGCGAGCGGC
TCGCATCTGCCCGCCACTCTGTCCCCCGAGACGATCAAGATCCTGCGCGAGGACCTCGCGTTCGAG
GGCGTCATCATGACCGAATGTCTGGAAATGGACGGCGTTCGAGCCGCATACGGAACCGTAGAGGGC
GCCCTCATGGCGCTCAAGGCGGGCGTCGACAATGTCATGATTTGCCACACGTACGACGTGCAAGCT
GCTTCCATCGACCGCGTCTGCGAGGCCGTCCATGCCGGAGAGCTGTCCCAGGCACGGCTCGACGCA
TCTCTCAAGCGGCTGCGCGACCTCAAGGACAAGTACACAAGCTGGGACACGGCACTGGAAGCCCGG
CCGCCATCGGACCTGGCCCGCCTGAGCACGGAGAACGAGGCCCTCGCCCACGACATTTACGCCAAC
GCCACCACGGTTGTCCGGTCGGAAGCAGGCCTCTTGCCCGTGTCCAGAAGCGCCAGCACGGTATTC
GTCTCGCCGGGCGTCAACGTTCCCACCAGCGGCGCCGCGTCCAGCGGGGAGGAGCTGCAGAAGACG
CGCGTGCCCTGGGTTTCCGGCGCCTTTGGCGACTCCCTCCGCAGGTACAACCCTGCTGTTGAGGAC
ATTCGCTTCACAGAGTCTACCTTGACGCCGGAGCAGTGGGGGCGGGTGGAGGACGCGGCCGTGGTC
GTTTTGGCGACGAGAAACGCGAGGGAGTCGCAGTACCAGCGGAGTCTGGGGCTGGAGATTGCCAGG
CGGCGGGCGGGCAGGACGCTCGTCGCGGTGGCTACCTGCAGTCCGTATGACTTTATCGACGACGAG
GCCGAGGTGAGGAATTATATTGCCGTGTATGAGCCTACTCTGGAGGCATTTGCGTCGGCGGCCGAC
ATTATTTACGGCGCGGCCACGGCCAAGGGTAGACTGCCTGTTGCTCACTAG

MaNAG4 amino acid sequence

MRPSIICDRDASRPLTGGVQPSTRNIKPAPTLIFPTDPACMGLFAVGFYGTEINKEIKSLIQDYGV
GAVLLFKRNIKDAAQLQALCLGLQQLAQDAGHTQPLFVGIDQENGLVTRISPPVAAQLPGPMALGA
AASLESAYHVAKATGNMLRYFGINMNYAPVGDVNNEPLNPVIGVRSPGDDADKVARFAAECAKGLR
ETRVAPCIKHFPGHGDTAVDSHYGLPVVNKTRGELEALELVPFRRAAAEGIEMVMTAHIALPKASG
SHLPATLSPETIKILREDLAFEGVIMTECLEMDGVRAAYGTVEGALMALKAGVDNVMICHTYDVQA
ASIDRVCEAVHAGELSQARLDASLKRLRDLKDKYTSWDTALEARPPSDLARLSTENEALAHDIYAN
ATTVVRSEAGLLPVSRSASTVFVSPGVNVPTSGAASSGEELQKTRVPWVSGAFGDSLRRYNPAVED
IRFTESTLTPEQWGRVEDAAVVVLATRNARESQYQRSLGLEIARRRAGRTLVAVATCSPYDFIDDE
AEVRNYIAVYEPTLEAFASAADIIYGAATAKGRLPVAH

Figure S3 - MaNAG4 nucleotide and amino acid sequence.
