# Supplementary material for: Molecular evolution and transcriptional profile of GH3 and GH20 β-N-acetylglucosaminidases in the entomopathogenic fungus Metarhizium anisopliae
Source: Genet Mol Biol. 2018 Dec 10;41(4):843–57. doi: 10.1590/1678-4685-GMB-2017-0363 (PMC6415606; doi:10.1590/1678-4685-GMB-2017-0363)
Supplement: Supplementary file 4 [file 1415-4757-GMB-1678-4685-GMB-2017-0363-s004.pdf]

Supplementary Material to "Molecular evolution and transcriptional profile of GH3 and GH20 β-N-acetylglucosaminidases in the entomopathogenic fungus Metarhizium anisopliae"

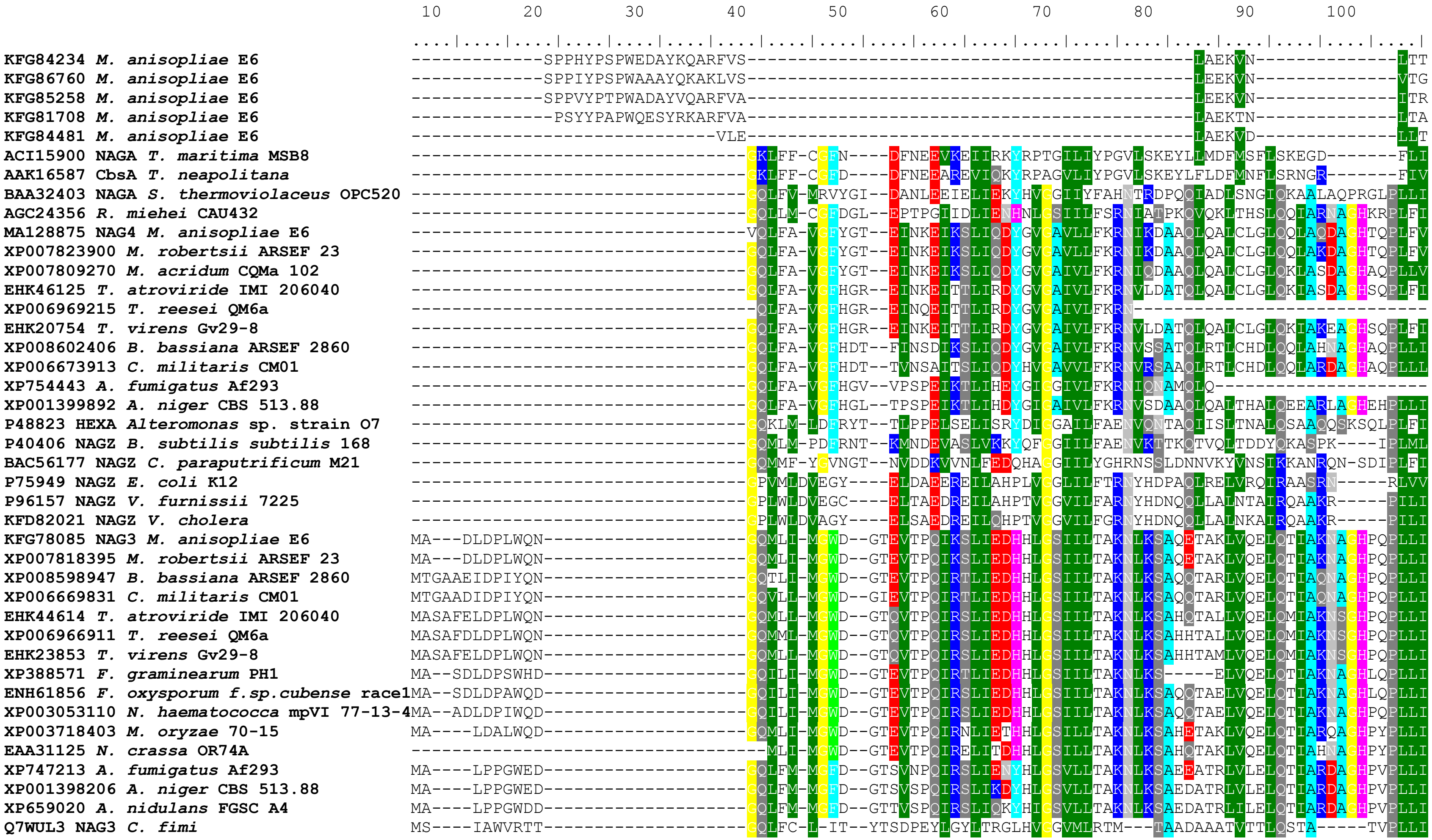

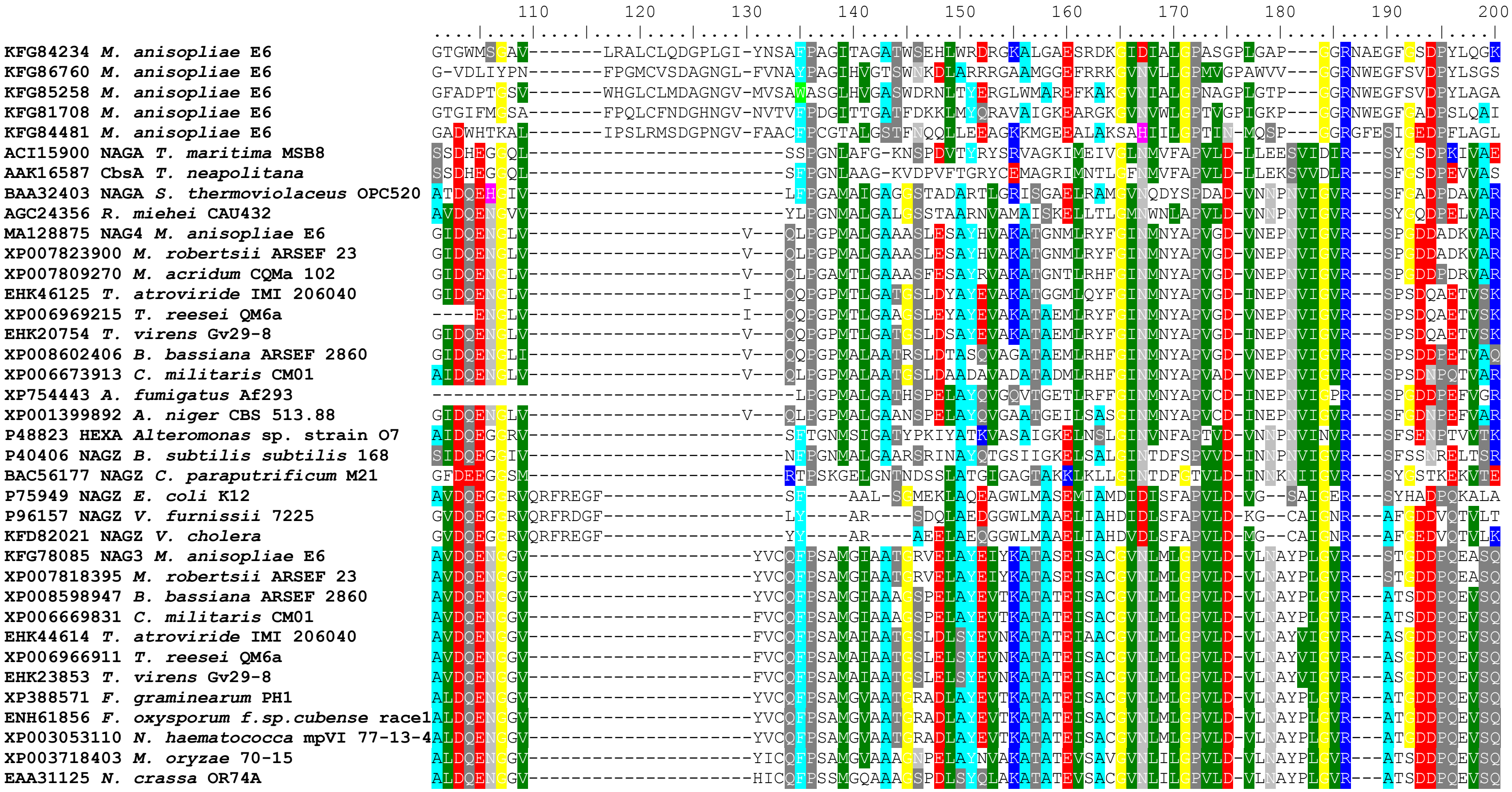
110 120 130 140 150 160 170 180 190 200
KFG84234 M. anisopliae E6 GTGWMSGAV-------LRALCLQDGPLGI-YNSAFFAGITAGATWSEHLWRDRGKALGAESRDKGIDIALGPASGPLGAP---GGRNAEGFGSDPYLQGK
KFG86760 M. anisopliae E6 G-VDLIYPN-------FPGMCVSDAGNGL-FVNAYPAGIHVGTSWNKDLARRRGAAMGGEFRRKGVNVLLGPMVGPAWVV---GGRNWEGFSVDPYLSGS
KFG85258 M. anisopliae E6 GFADPTGSV-------WHGLCLMDAGNGV-MVSAWASGLHVGASWDRNLTYERGLWMAREFKAKGVNIALGPNAGPLGTP---GGRNWEGFSVDPYLAGA
KFG81708 M. anisopliae E6 GTGIFMGSA-------FPQLCFNDGHNGV-NVTVFPDGITTGATFDKKLMYQRAVAIGKEARGKGVNVWLGPTVGPIGKP---GGRNWEGFGADPSLQAI
KFG84481 M. anisopliae E6 GADWHTKAL-------IPSLRMSDGPNGV-FAACFPCGTALGSTFNQQLLEEAGKKMGEEALAKSAHIILGPTIN-MQSP---GGRGFESIGEDPFLAGL
ACI15900 NAGA T. maritima MSB8 SSDHEGGQL----------------------SSPGNLAFG-KNSPDVTYRYSRVAGKIMEIVGLNMVFAPVLD-LLEESVIDIR---SYGSDPKIVAE
AAK16587 CbsA T. neapolitana SSDHEGGQL----------------------SFPGNLAAG-KVDPVFTGRYCEMAGRIMNTLGFNMVFAPVLD-LLEKSVVDLR---SFGSDPEVVAS
BAA32403 NAGA S. thermoviolaceus OPC520 ATDQEHGIV----------------------LFPGAMAIGAGGSTADARTLGRISGAELRAMGVNQDYSPDAD-VNNPNVIGVR---SFGADPDAVAR
AGC24356 R. miehei CAU432 AVDQENGVV----------------------YLPGNMALGALGSSTAARNVAMAISKELLTLGMNWNLAPVLD-VNNPNVIGVR---SYGQDPELVAR
MA128875 NAG4 M. anisopliae E6 GIDQENGLV------------------V---QLPGPMALGAAASLESAYHVAKATGNMLRYFGINMNYAPVGD-VNEPNVIGVR---SPGDDADKVAR
XP007823900 M. robertsii ARSEF 23 GIDQENGLV------------------V---QLPGPMALGAAASLESAYHVAKATGNMLRYFGINMNYAPVGD-VNEPNVIGVR---SPGDDADKVAR
XP007809270 M. acridum CQMa 102 GIDQENGLV------------------V---QLPGAMTLGAAASFESAYRVAKATGNTLRHFGINMNYAPVGD-VNEPNVIGVR---SPGDDPDRVAR
EHK46125 T. atroviride IMI 206040 GIDQENGLV------------------I---QQPGPMTLGATGSLDYAYEVAKATGGMLQYFGINMNYAPVGD-INEPNVIGVR---SPSDQAETVSK
XP006969215 T. reesei QM6a ----ENGLV------------------I---QQPGPMTLGAAGSLEYAYEVAKATAEMLRYFGINMNYAPVGD-VNEPNVIGVR---SPSDQAETVSK
EHK20754 T. virens Gv29-8 GIDQENGLV------------------V---QQPGPMTLGATGSLDSAYEVAKATAEMLRYFGINMNYAPVGD-INEPNVIGVR---SPSDQAETVSK
XP008602406 B. bassiana ARSEF 2860 GIDQENGLI------------------V---QQPGPMALAATRSLDTASQVAGATAEMLRHFGINMNYAPVGD-VNEPNVIGVR---SPSDDPETVAQ
XP006673913 C. militaris CM01 AIDQENGLV------------------V---QLPGPMALAATGSLDAADAVADATADMLRHFGINMNYAPVAD-VNEPNVIGVR---SPSDNPQTVAR
XP754443 A. fumigatus Af293 ---------------------------------LPGPMALGATHSPELAYQVGGQVTGETLRFFGINMNYAPVCD-INEPNVIGPR---SPGDDPEFVGR
XP001399892 A. niger CBS 513.88 GIDQENGLV------------------V---QLPGPMALGAANSPELAYQVGAATGEILSASGINMNYAPVCD-INEPNVIGVR---SFGDNPEFVAR
P48823 HEXA Alteromonas sp. strain O7 AIDQEGGRV----------------------SFTGNMSIGATYPKIYATKVASAIGKEINSLGINVNFAPTVD-VNNPNVINVR---SFSENPTVVTK
P40406 NAGZ B. subtilis subtilis 168 SIDQEGGIV----------------------NEPGNMALGAARSRINAYQTGSIIGKELSALGINTDFSPVVD-INNPNVIGVR---SFSSNRELTSR
BAC56177 NAGZ C. paraputrificum M21 GFDEEGGSM----------------------RTPSKGELGNTNDSSLATGIGAGTAKKLKLLGINTDFGTVLD-INNKNIIGVR---SYGSTKEKVTE
P75949 NAGZ E. coli K12 AVDQEGGRVQRFREGF-----------------SF----AAL-SGMEKLAQEAGWLMASEMIAMDIDISFAPVLD-VG--SAIGER---SYHADPQKALA
P96157 NAGZ V. furnissii 7225 GVDQEGGRVQRFRDGF-----------------LY----AR----SDQLAEDGGWLMAAELIAHDIDLSFAPVLD-KG--CAIGNR---AFGDDVQTVLT
KFD82021 NAGZ V. cholera GVDQEGGRVQRFREGF-----------------YY----AR----AEELAEQGGWLMAAELIAHDVDLSFAPVLD-MG--CAIGNR---AFGEDVQTVLK
KFG78085 NAG3 M. anisopliae E6 AVDQENGGV--------------------YVCQFPSAMGIAATGRVELAYEIYKATASEISACGVNLMLGPVLD-VLNAYPLGVR---STGDDPQEASQ
XP007818395 M. robertsii ARSEF 23 AVDQENGGV--------------------YVCQFPSAMGIAATGRVELAYEIYKATASEISACGVNLMLGPVLD-VLNAYPLGVR---STGDDPQEASQ
XP008598947 B. bassiana ARSEF 2860 AVDQENGGV--------------------YVCQFPSAMGIAAAGSPELAYEVTKATATEISACGVNLMLGPVLD-VLNAYPLGVR---ATSDDPQEVSQ
XP006669831 C. militaris CM01 AVDQENGGV--------------------FVCQFPSAMGIAAAGSPELAYEVTKATATEISACGVNLMLGPVLD-VLNAYPLGVR---ATSDDPQEVSQ
EHK44614 T. atroviride IMI 206040 AVDQENGGV--------------------FVCQFPSAMAIAATGSLDLSYEVNKATATEIAACGVNLMLGPVLD-VLNAYVIGVR---ASGDDPQEVSQ
XP006966911 T. reesei QM6a AVDQENGGV--------------------FVCQFPSAMAIAATGSLELSYEVNKATATEISACGVNLMLGPVLD-VLNAYVIGVR---ASGDDPQEVSQ
EHK23853 T. virens Gv29-8 AVDQENGGV--------------------FVCQFPSAMAIAATGSLELSYEVNKATATEISACGVNLMLGPVLD-VLNAYVIGVR---ASGDDPQEVSQ
XP388571 F. graminearum PH1 ALDQENGGV--------------------YVCQFPSAMGVAATGRADLAYEVTKATATEISACGVNLMLGPVLD-VLNAYPLGVR---ATGDDPQEVSQ
ENH61856 F. oxysporum f.sp.cubense race1 ALDQENGGV--------------------YVCQFPSAMGVAATGRADLAYEVTKATATEISACGVNLMLGPVLD-VLNAYPLGVR---ATGDDPQEVSQ
XP003053110 N. haematococca mpVI 77-13-4 ALDQENGGV--------------------YVCQFPSAMGVAATGRADLAYEVTKATATEISACGVNLMLGPVLD-VLNAYPLGVR---ATGDDPQEVSQ
XP003718403 M. oryzae 70-15 ALDQENGGV--------------------YICQFPSAMGVAAAGNPELAYNVAKATATEVSAVGVNLILGPVLD-VLNAYPLGVR---ATSDDPQEVSQ
EAA31125 N. crassa OR74A ALDQENGGV--------------------HICQFPSSMGQAAAGSPDLSYQLAKATATEVSACGVNLILGPVLD-VLNAYPLGVR---ATSDDPQEVSQ

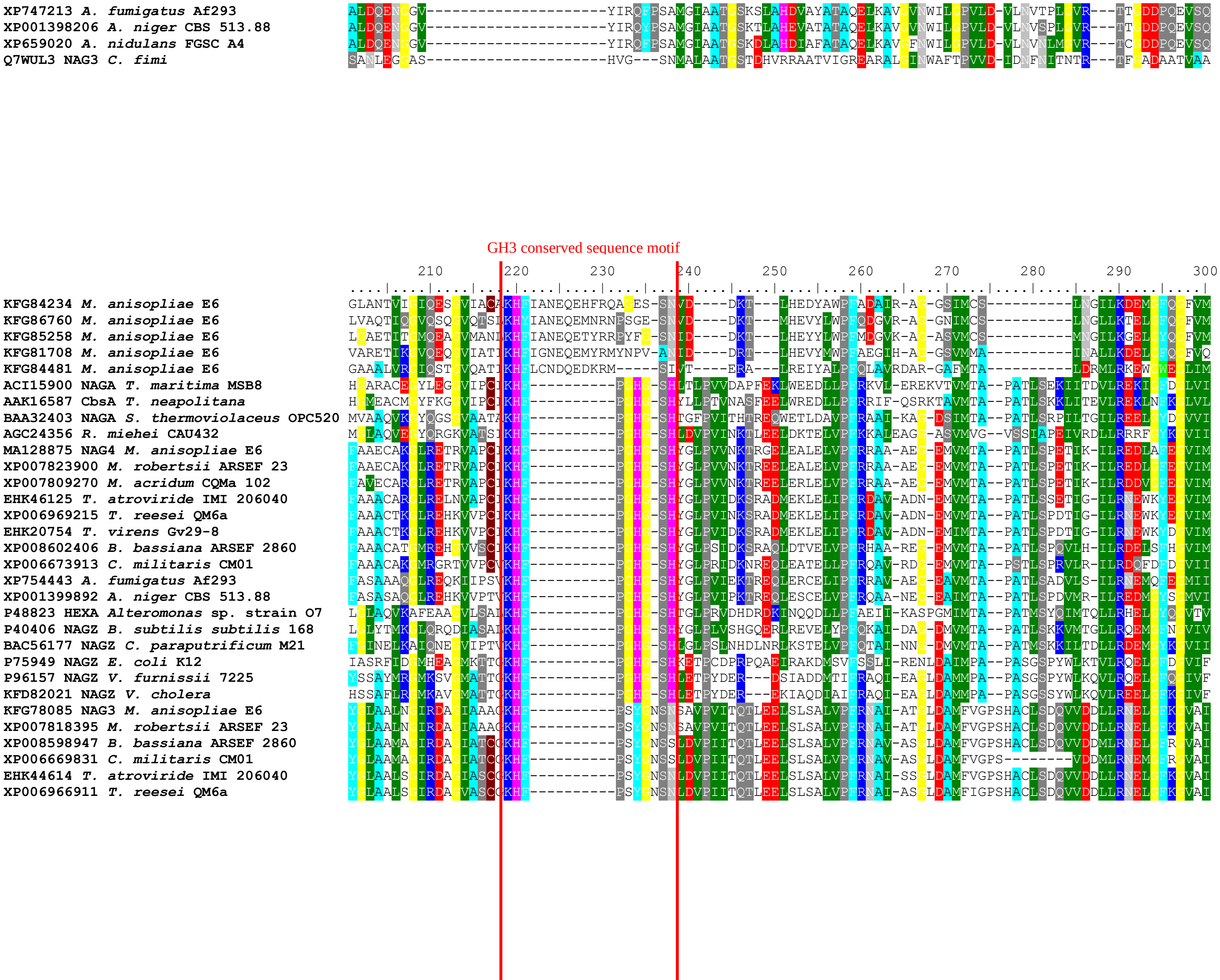
XP747213 A. fumigatus Af293
XP001398206 A. niger CBS 513.88
XP659020 A. nidulans FGSC A4
Q7WUL3 NAG3 C. fimi
GH3 conserved sequence motif
210
220
230
240
250
260
270
280
290
300
KFG84234 M. anisopliae E6
KFG86760 M. anisopliae E6
KFG85258 M. anisopliae E6
KFG81708 M. anisopliae E6
KFG84481 M. anisopliae E6
ACI15900 NAGA T. maritima MSB8
AAK16587 CbsA T. neapolitana
BAA32403 NAGA S. thermoviolaceus OPC520
AGC24356 R. miehei CAU432
MA128875 NAG4 M. anisopliae E6
XP007823900 M. robertsii ARSEF 23
XP007809270 M. acridum CQMa 102
EHK46125 T. atroviride IMI 206040
XP006969215 T. reesei QM6a
EHK20754 T. virens Gv29-8
XP008602406 B. bassiana ARSEF 2860
XP006673913 C. militaris CM01
XP754443 A. fumigatus Af293
XP001399892 A. niger CBS 513.88
P48823 HEXA Alteromonas sp. strain O7
P40406 NAGZ B. subtilis subtilis 168
BAC56177 NAGZ C. paraputrificum M21
P75949 NAGZ E. coli K12
P96157 NAGZ V. furnissii 7225
KFD82021 NAGZ V. cholera
KFG78085 NAG3 M. anisopliae E6
XP007818395 M. robertsii ARSEF 23
XP008598947 B. bassiana ARSEF 2860
XP006669831 C. militaris CM01
EHK44614 T. atroviride IMI 206040
XP006966911 T. reesei QM6a

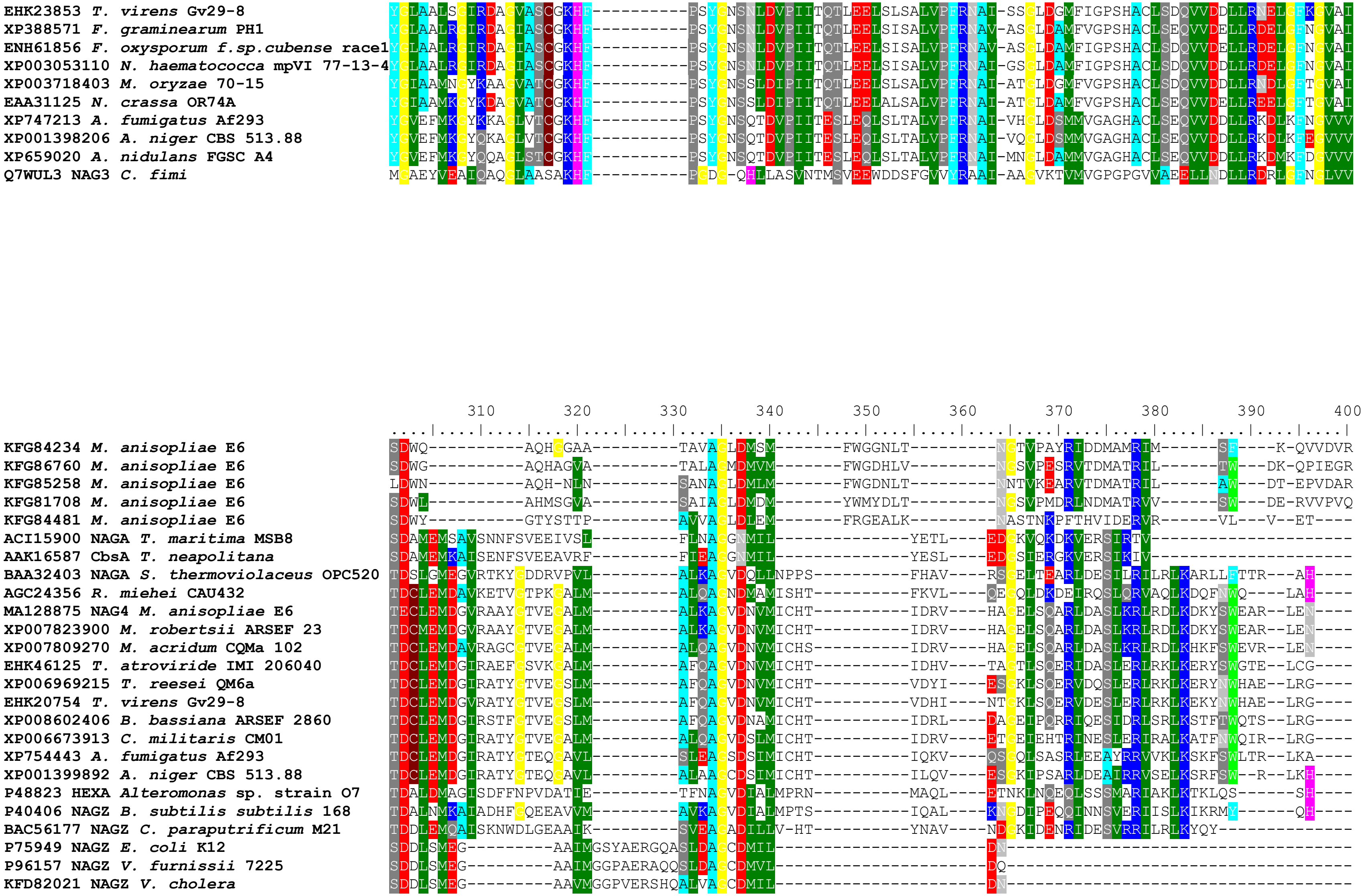
EHK23853 T. virens Gv29-8
XP388571 F. graminearum PH1
ENH61856 F. oxysporum f.sp.cubense race1
XP003053110 N. haematococca mpVI 77-13-4
XP003718403 M. oryzae 70-15
EAA31125 N. crassa OR74A
XP747213 A. fumigatus Af293
XP001398206 A. niger CBS 513.88
XP659020 A. nidulans FGSC A4
Q7WUL3 NAG3 C. fimi
310 320 330 340 350 360 370 380 390 400
KFG84234 M. anisopliae E6
KFG86760 M. anisopliae E6
KFG85258 M. anisopliae E6
KFG81708 M. anisopliae E6
KFG84481 M. anisopliae E6
ACI15900 NAGA T. maritima MSB8
AAK16587 CbsA T. neapolitana
BAA32403 NAGA S. thermoviolaceus OPC520
AGC24356 R. miehei CAU432
MA128875 NAG4 M. anisopliae E6
XP007823900 M. robertsii ARSEF 23
XP007809270 M. acridum CQMa 102
EHK46125 T. atroviride IMI 206040
XP006969215 T. reesei QM6a
EHK20754 T. virens Gv29-8
XP008602406 B. bassiana ARSEF 2860
XP006673913 C. militaris CM01
XP754443 A. fumigatus Af293
XP001399892 A. niger CBS 513.88
P48823 HEXA Alteromonas sp. strain O7
P40406 NAGZ B. subtilis subtilis 168
BAC56177 NAGZ C. paraputrificum M21
P75949 NAGZ E. coli K12
P96157 NAGZ V. furnissii 7225
KFD82021 NAGZ V. cholera

```
KFG78085 NAG3 M. anisopliae E6            SECLEMEALSHDLGVQNGVVM---------AVEAGCDLVL----RAY-----------KLGYENGIITKDRIFTSIRRVQHLKSTCSW---P--SH----
XP007818395 M. robertsii ARSEF 23         SECLEMEALSHDLGVQNGVVM---------AVEAGCDLVL----RAY-----------KLGYENGIITKDRIFTSIRRVQHLKSTCSW---P--SH----
XP008598947 B. bassiana ARSEF 2860        SECLEMEALSHDLGVQNGVVM---------AVEAGCDLVL----RAY-----------KLGLENGIITKERIFTSLRRVLHLKTSCSW---P--SH----
XP006669831 C. militaris CM01             SECLEMEALSHDLGVQNGVVM---------AVEAGCDLVL----RAY-----------KLGLENGIITKERLFTSVRRVLHLKSNCSW---P--SH----
EHK44614 T. atroviride IMI 206040        SECLEMEALSQDLGVQNGVVM---------AVEAGCDIIL----RAY-----------KLGYENGIITKERIFTSLRRIFHLKSTCSW---P--SH----
XP006966911 T. reesei QM6a               SECLEMEALSQDLGVQNGVVM---------AVEAGCDIVL----RAY-----------KLGYENGIITKERIFTSLKRIFHLKSTCSW---P--SH----
EHK23853 T. virens Gv29-8                SECLEMEALSQDLGVQNGVVM---------AVEAGCDIVL----RAY-----------KLGYENGIVTKERIFTSLRRIFHLKSSCSW---P--SH----
XP388571 F. graminearum PH1              SECLEMEALSHELGVQNGVIM---------AVEAGCDLVL----RAY-----------KLGYDNGIVSKERIFTSLRRVLNLKSTCSW---P--SH----
ENH61856 F. oxysporum f.sp.cubense race1 SECLEMEALSHELGVQNGVIM---------AVEAGCDLVL----RAY-----------KL----------------------------------------
XP003053110 N. haematococca mpVI 77-13-4 SECLEMEALSHELGVQNGVVM---------AVEAGCDLVL----RAY-----------KLGYENGIITKERIFTSLRRVLQLKSTCSW---P--SH----
XP003718403 M. oryzae 70-15               SECLEMEALSHEIGVKGGTVM---------AVEAGCDLVL----RAY-----------KLGIENGIITRERIYTSLQRVLRMKRGCSW---P--SH----
EAA31125 N. crassa OR74A                 SECLEMEALRTEMGVRTGTIM---------AVQAGCDLVL----RAY-----------KLGLENEVLTKERVYTSLRRVLKMKRGCDW---P--SH----
XP747213 A. fumigatus Af293              SECLEMEALTHNIGVGGGTVM---------AKNAGCDIIL----RSF-----------KLGVENGIIGRARIEQSLRRVLKMKAKCSW---P--SH----
XP001398206 A. niger CBS 513.88          SECLEMEALTHNIGVGGGTVM---------AKNAGCDIIL----RSF-----------KLGVENGIIGRARIEQSLRRVLKLKAKCSW---P--SH----
XP659020 A. nidulans FGSC A4             SECLEMEALTHNIGVGGGTVM---------AKNAGCDIIL----RSF-----------KLGVENGIINRTRIEESLRRVLAMKGRCSW---P--SH----
Q7WUL3 NAG3 C. fimi                      SDSTTMAGLASVLPRSQAVPR---------VIAAGCDMFL----KNL-----------RAGIRDGVITPERLDEAVTRILALKA----------DH----
```

```
                                                 410       420       430       440       450       460       470       480       490       500
                                         ....|....|....|....|....|....|....|....|....|....|....|....|....|....|....|....|....|....|....|....|
KFG84234 M. anisopliae E6                AAGTVILK-------------------------------------------------SLP----------------------------------------
KFG86760 M. anisopliae E6                DRGHVLVK------------------------------------------------NTLP----------------------------------------
KFG85258 M. anisopliae E6                SKGHVLVK------------------------------------------------HALP----------------------------------------
KFG81708 M. anisopliae E6                NEAITLLK------------------------------------------------NLLP----------------------------------------
KFG84481 M. anisopliae E6                AASIVLLK------------------------------------------------NVLP----------------------------------------
ACI15900 NAGA T. maritima MSB8          -GAVEFLGFEKIDH---------------------------------------V----------------------------------------------
AAK16587 CbsA T. neapolitana            -GAIECTRMRKELL---------------------------------------I----------------------------------------------
BAA32403 NAGA S. thermoviolaceus OPC520 LRTTTLLVNKGNLLPLSRR------------------AALNELGFTALTDQAVVVA--------------------------------------------
AGC24356 R. miehei CAU432              ATVPTVVTNRKNTLPIRPAVPQTLAVDS---------TNLEYIIYN--TPDLSIIG--------------------------------------------
MA128875 NAG4 M. anisopliae E6           EAATTVVRSEAGLLPVSRSVPTSGAASSQKTRVPWVSPAVEDIRFTTLTPEQWVLA--------------------------------------------
XP007823900 M. robertsii ARSEF 23       EAATTVVRSEAGLLPVSRTVPTSGAASSQKTRVPWVSPAVEDIRFTTLTPEQWVLA--------------------------------------------
XP007809270 M. acridum CQMa 102        EAATTVVRSEAGLLPVSRTVVTSAAASSHKTRVPWVSPAIADVPFASLTPEQWILA--------------------------------------------
EHK46125 T. atroviride IMI 206040      EKAVTLVRAEEGLLPLKPTVPIGGAVDSLPTRVPWIASQTTDIRFTTLTDEQWILA--------------------------------------------
XP006969215 T. reesei QM6a             EKATTLVRAQEGLLPLKATVPIGGAVDSLPTRVPWIAPEMSDVRFTNLTEEQWILA--------------------------------------------
EHK20754 T. virens Gv29-8              EKATTLVRAQEGLLPLKSTVPIGGAVDSLPTRVPWIAPQFSDVRFITLTDEQWILA--------------------------------------------
XP008602406 B. bassiana ARSEF 2860      AKAATVVRIEDGVIPVSKSFPVGGAVNSHPTRVPWVSAVAEEIRYTPLTDEQWILA--------------------------------------------
XP006673913 C. militaris CM01           AASATVVRVQPGVFPVSPSVPVGGAVNSQPTRVPWVSAALEEIRYTPLSDAQWLLA--------------------------------------------
XP754443 A. fumigatus Af293            ATSVTLVRSDPDVLPLSKSTPAGGAVDA---------QATVELHYGELSTEQYVLV--------------------------------------------
XP001399892 A. niger CBS 513.88        SASVTLVRDSLQILPVSSSTPAGGAVDS---------QTVVEIRYGGLSAELTIFT--------------------------------------------
```

**P48823 HEXA *Alteromonas* sp. strain O7**	RRAITEVKNDG-VLPLRDN----------------------------------IAA--
**P40406 NAGZ *B. subtilis subtilis* 168**	LKAVTVLKNEQHTLPFKPK----------------------------------ITG--
**BAC56177 NAGZ *C. paraputrificum* M21**	--ANN---
**P75949 NAGZ *E. coli* K12**	--VTRLYH--
**P96157 NAGZ *V. furnissii* 7225**	--AQSLLK--
**KFD82021 NAGZ *V. cholera***	--AEVLMK--
**KFG78085 NAG3 *M. anisopliae* E6**	VSSITVIR--KMLPLASSMHPGEELLLLTPLVKPLPASAMTKSLLESKKTPTQH
**XP007818395 *M. robertsii* ARSEF 23**	VSSITVIR--KMLPLSSSMHPGEELLLLTPLVKPLPASAMTKSLLESKKTPTQH
**XP008598947 *B. bassiana* ARSEF 2860**	LTSITVIR--QLLPLSQSMHPGEELLLLTPLVKPLPASSLTKSLLDAKQDTGNH
**XP006669831 *C. militaris* CM01**	LASITVIR--QLLPLSQSVHPGEELLLLTPLVKPLPASSLTKSFLDSKQDMGNH
**EHK44614 *T. atroviride* IMI 206040**	LASITIVR--KLLPLSLSMHPGEELLLLTPLVKPLPASSLTKSLTEARNDSTHH
**XP006966911 *T. reesei* QM6a**	LASITIVR--KLLPLSLSMHPGEELLLLTPLVKPLPASSLTKSLLESKNDSTEH
**EHK23853 *T. virens* Gv29-8**	LASITIVR--KLLPLSLSLHPGEELLLLTPLVKPLPASSLTKSLLESKNDTTMH
**XP388571 *F. graminearum* PH1**	LASITIIR--KLIPLTASMHPGEELLLLTPLVKPLPASSLTKKLLAAKDSEGPH
**ENH61856 *F. oxysporum f.sp.cubense* race1**	--ASMHPGEELLLLTPLVKPLPASSLTKKLLAAKDSEGQH
**XP003053110 *N. haematococca* mpVI 77-13-4**	LASITVIR--KLLPLSASMHPGEELLLLTPLVKPLPASSLTKKLLSTKGDWGSH
**XP003718403 *M. oryzae* 70-15**	LASITVMR--KLLPLNGSMHQDDELLLLTPLVKPLPASAATKTLLRSKDTSGVH
**EAA31125 *N. crassa* OR74A**	LASITVMR--KLLPLNESMHQEEELLLLTPLVKPLPASAMTKTILEGSTKNPIH
**XP747213 *A. fumigatus* Af293**	TSSISVVR--NLLPLSNIIEPNEELLLLTPLVKPLPASAVSRSVTEHMNL----
**XP001398206 *A. niger* CBS 513.88**	TSSISVVR--NLLPLSNILESNEELLLLTPLVKPLPASAVSRSVSEHSNM----
**XP659020 *A. nidulans* FGSC A4**	TSSISVVR--NLLPLTNVLSSNEELLLLTPLVNPLPASAVSRSVTEHLEL----
**Q7WUL3 NAG3 *C. fimi***	SASITLVK--GVLP--

	510 520 530 540 550 560 570 580 590 600
	....|....|....|....|....|....|....|....|....|....|....|....|....|....|....|....|....|....|....|....|
**KFG84234 *M. anisopliae* E6**	--
**KFG86760 *M. anisopliae* E6**	--
**KFG85258 *M. anisopliae* E6**	--
**KFG81708 *M. anisopliae* E6**	--
**KFG84481 *M. anisopliae* E6**	--
**ACI15900 NAGA *T. maritima* MSB8**	--
**AAK16587 CbsA *T. neapolitana***	--
**BAA32403 NAGA *S. thermoviolaceus* OPC520**	--
**AGC24356 *R. miehei* CAU432**	--
**MA128875 NAG4 *M. anisopliae* E6**	--
**XP007823900 *M. robertsii* ARSEF 23**	--
**XP007809270 *M. acridum* CQMa 102**	--
**EHK46125 *T. atroviride* IMI 206040**	--

```
XP006969215 T. reesei QM6a                  ----------------------------------------------------------------------------------------------------
EHK20754 T. virens Gv29-8                   ----------------------------------------------------------------------------------------------------
XP008602406 B. bassiana ARSEF 2860          ----------------------------------------------------------------------------------------------------
XP006673913 C. militaris CM01                ----------------------------------------------------------------------------------------------------
XP754443 A. fumigatus Af293                 ----------------------------------------------------------------------------------------------------
XP001399892 A. niger CBS 513.88             ----------------------------------------------------------------------------------------------------
P48823 HEXA Alteromonas sp. strain O7       ----------------------------------------------------------------------------------------------------
P40406 NAGZ B. subtilis subtilis 168        ----------------------------------------------------------------------------------------------------
BAC56177 NAGZ C. paraputrificum M21         ----------------------------------------------------------------------------------------------------
P75949 NAGZ E. coli K12                     ----------------------------------------------------------------------------------------------------
P96157 NAGZ V. furnissii 7225               ----------------------------------------------------------------------------------------------------
KFD82021 NAGZ V. cholera                    ----------------------------------------------------------------------------------------------------
KFG78085 NAG3 M. anisopliae E6              DSWKDIMSGEGVF---------------------------LHENLIARASCIIIVTADANRNMYQAGFTKHVDMMCSMHRSRGNKKQLIVVAVSSPYDFA
XP007818395 M. robertsii ARSEF 23           DSWKDIMSGEGVF---------------------------LHENLIARASCIIIVTADANRNMYQAGFTKHVDMMCSMHRSRGNKKQLIVVAVSSPYDFA
XP008598947 B. bassiana ARSEF 2860          ERWRRHMSGEGVFREFGKTLARYRNEKLLHTSYTANGVRPVHENLINRASCIIIFTADANRNLYQAGFTKHVDMMCSMLRSRGQKKHLIVVAVSSPYDFA
XP006669831 C. militaris CM01               DRWRRHMSGEGVFREFGKTLARHRNEKLLHTSYTANGVRPVHENLINRASCIIIFTADANRNLYQAGFTKHVDMMCSMLRSRGQKKHLIVVAVSSPYDFA
EHK44614 T. atroviride IMI 206040           DKWQGIMSGEGVFREFGKTLARYRNEKLLHTSYTANGVRPVHENLINRASCIVIFTADANRNLYQAGFTKHVDMMCSMLRSRGQKKQLIVVAVSSPYDFA
XP006966911 T. reesei QM6a                  DRWQIIMSGEGVFREFGKTLARYRNEKLLHTSYTANGVRPVHENLINRASCIIIFTADANRNLYQAGFTKHVDMMCSMLRSRGQKKQLIVVAVSSPYDFA
EHK23853 T. virens Gv29-8                   DKWQIIMSGEGVFREFGKTLARYRNEKLLHTSYTANGVRPVHENLINRASCIIIFTADANRNLYQAGFTKHVDMMCSMLRSRGQKKQLIVVAVSSPYDFA
XP388571 F. graminearum PH1                 EMWNGILSGEGVFREFGKSLARARNEKLLHTSYTANGVRPVHENLIHRASCIVIVTADANRNLYQAGFTKHVDMMCSMLRARGQKKQLIVVAVSSPYDFA
ENH61856 F. oxysporum f.sp.cubense race1EMWNGILSGEGVFREFGKSLARARNEKLLHTSYTANGVRPVHENLIHRASCIIIVTADANRNLYQAGFTKHVDMMCSMLRTRGQKKQLIVVAVSSPYDFA
XP003053110 N. haematococca mpVI 77-13-4HIWNDIMSGEGVFREFGKSLARARNEKLLHTSYTANGVRPVHENLIHRASCIIIVTADANRNLYQAGFTKHVDMMCAMLRSRGQKKQLIVVAVSSPYDFA
XP003718403 M. oryzae 70-15                 DKW--LMSGEGVFREFGRSLARARHGKLLHTSYTANGVRPVHENLIDKASCIIIVTADANRNLYQAGFTKHVATICAMLRAGGQKKSLIVVAVSSPYDFA
EAA31125 N. crassa OR74A                   DKW--IMSGEGVFRELGRSLARARHGKLLHTSYTANGVRPVHESLIQRASTIILLTADANRNLYQAGFTKHVAMMCSLLKASGHKKNLIVVAVSSPYDFA
XP747213 A. fumigatus Af293                 -----VLSGESVFKELGRSLSRQRSGRVLHTSYTANGVRPIHENLIDRASAVIVVTADANRNLYQHGFTKHVSLICSQFSPSGEEKPMIVIAASSPYDFA
XP001398206 A. niger CBS 513.88             -----VLSGESVFKELGRSLSRQRSGRVLHTSYTANGVRPIHENLIDRANAVIVVTADANRNLYQHGFTKHVSMICSQFTPSGEEKPLIVVAASSPYDFA
XP659020 A. nidulans FGSC A4                -----VLSGESVFKELGRSLSRHRNGRVLHTSYTSNGVRPIHESLIDRASAVIVITADAVRNIYQQGFTKHVSMICSQLTPSGEDKPLVVVAVSSPYDFA
Q7WUL3 NAG3 C. fimi                        ----------------------------------------------------------------------------------------------------
```

```
                                                     610       620       630       640       650       660       670       680       690       700
                                            ....|....|....|....|....|....|....|....|....|....|....|....|....|....|....|....|....|....|....|....|
KFG84234 M. anisopliae E6                   -----------------------GED--------------------------------------------------------------------------
KFG86760 M. anisopliae E6                   -----------------------GYSPGDL--LINLWR--------------------------------------------------------------
KFG85258 M. anisopliae E6                   -----------------------GYD--DV--LFQL----------------------------------------------------------------
KFG81708 M. anisopliae E6                   -----------------------GTG--------------------------------------------------------------------------
KFG84481 M. anisopliae E6                   -----------------------GPNSASLPAYYAVTP--------------------------------------------------------------
ACI15900 NAGA T. maritima MSB8             ----------------------------------------------------------------------------------------------------
AAK16587 CbsA T. neapolitana               ----------------------------------------------------------------------------------------------------
```

```
BAA32403 NAGA S. thermoviolaceus OPC520 ----------------------------------------------------------------------------------------------------
AGC24356 R. miehei CAU432                ----------------------------------------------------------------------------------------------------
MA128875 NAG4 M. anisopliae E6          ----------------------------------------------------------------------------------------------------
XP007823900 M. robertsii ARSEF 23       ----------------------------------------------------------------------------------------------------
XP007809270 M. acridum CQMa 102         ----------------------------------------------------------------------------------------------------
EHK46125 T. atroviride IMI 206040       ----------------------------------------------------------------------------------------------------
XP006969215 T. reesei QM6a              ----------------------------------------------------------------------------------------------------
EHK20754 T. virens Gv29-8               ----------------------------------------------------------------------------------------------------
XP008602406 B. bassiana ARSEF 2860      ----------------------------------------------------------------------------------------------------
XP006673913 C. militaris CM01           ----------------------------------------------------------------------------------------------------
XP754443 A. fumigatus Af293             ----------------------------------------------------------------------------------------------------
XP001399892 A. niger CBS 513.88         ----------------------------------------------------------------------------------------------------
P48823 HEXA Alteromonas sp. strain O7   ----------------------------------------------------------------------------------------------------
P40406 NAGZ B. subtilis subtilis 168    ----------------------------------------------------------------------------------------------------
BAC56177 NAGZ C. paraputrificum M21     ----------------------------------------------------------------------------------------------------
P75949 NAGZ E. coli K12                 ----------------------------------------------------------------------------------------------------
P96157 NAGZ V. furnissii 7225           ----------------------------------------------------------------------------------------------------
KFD82021 NAGZ V. cholera                ----------------------------------------------------------------------------------------------------
KFG78085 NAG3 M. anisopliae E6          MDKSIGTYICTFDFTEDAMSALV---PGTLRKSKKVLKSRQHWLVEEYDRARDYAGLEDLLKAVHRASAPDLHFLKSTRAASMELHLPNIKETHFVVRNS
XP007818395 M. robertsii ARSEF 23       MDKSIGTYICTFDFTEDAMSALV---PGTLRKSKKVLKSRQHWLVEEYDRARDYGGLEDLLKAVHRASAPDLHFLKSTRAASMELHLPNIKETHFVVRNS
XP008598947 B. bassiana ARSEF 2860      MDKSIGTYICTFDFTENAMAALV---PGTLRKSKKVLKSRQLWLVEEYDRERDAAGLDDLLKAIHRSGGSDLDFLSNTGASAFELFNSNIKEAHYVVRNS
XP006669831 C. militaris CM01           MDKSIGTYICTFDFTENAMASLV---PGTLRKSKKVLKSRQLWLVEEYDRERDATGLDDLLKAIHRAGGSDLDFLSNTSASAFELFNNNIKEAHFVVRNS
EHK44614 T. atroviride IMI 206040       MDKSIGTYICTYDFTENAMAALV---PGTLRKSKKVLKSRQHWLVEEFDSTRDRNGLNDLIRAVHRASDQDMRYLQTTSAETFALNNPNVKEAHFVVRNS
XP006966911 T. reesei QM6a              MDKSIGTYICTYDFTENAMAALV---PGTLRKSKKVLKSRQHWLVEEFDSSRDRKGLNDLIRAVHRASDQDFRYLQTATADTFLLANQNIKETHFVVRNS
EHK23853 T. virens Gv29-8               MDKSIGTYICTYDFTENAMAALV---PGTLRKSKKVLKSRQHWLVEEFDSNRDRKGLNDLLRAVHRASDQDFRYLQTTTSDTFVLDNQNVKETHFVVRNS
XP388571 F. graminearum PH1             MDKSIGTYICTFDFTENALHALA---PGTLRKSKKVVKSRQHWLVEEYSSGRDASGLNDLLRAVHRASAPDLQFLRATTAASFQLNNPSIAESHFVVRNS
ENH61856 F. oxysporum f.sp.cubense race1MDKSIGTYLCTFDFTENALHALA---PGTLRKSKKVLKSRQHWLVEEYSAKRDASALNDLLRAVHRASAPDLQFLRTTTAASFQLNNANIAESHFVVRNS
XP003053110 N. haematococca mpVI 77-13-4MDKSIGTYLCTFDFTENALHALA---PGTLRKSKKVLKSRQHWLVEEYHTDRDASGLEDLIRAIHRASAPDLQFLRTTTAATFQLKNPNIAEAHVVVRNS
XP003718403 M. oryzae 70-15             MDKTIGTYICTFDFTETAMHALV---PGTLRKSKKVLQSRQQWLVESYERERDAHGLDDLIQSVARSSASN--HIAAATAATFELSNPRVDEAHFVVRNS
EAA31125 N. crassa OR74A               MDKSIGTYICTFDFTETAMSALV---PGTLRKARKAVQSSQHWLVEPYDVERDTKGLQDLLFAMVRGSAPNHQYFYAFGPHSFSLEEPKMEEQHFVVRNS
XP747213 A. fumigatus Af293             MDTSIGTYVCTYDFTETALEALV---PGSISRSQKLHQARQHWLVENWNEERDAQSLDTLLDAVRSE-------LLGVTSSSFLLRSEEIDEAHFVVRNS
XP001398206 A. niger CBS 513.88         MDPSIGTYICTYDFTETALEALV---PGSISRSQKLHQARQHWLVETWNEERDARSLDSLLDAVRSE-------LLGVTSSSFLLRREEIDEAHFVVRNS
XP659020 A. nidulans FGSC A4           MDASIGTYLCTYDFTDTALETLV---PGSFNRSQKLHQARQHWLVENWNEERDSDALDALLKTMGPE-------LSGVTPSSFLLRRDDIDEAHFVVRNS
Q7WUL3 NAG3 C. fimi                     --------------------------IGQGARAGAVEQ--------------------------------------------------------------
```

```
                                               710       720       730       740       750       760       770       780       790       800
                                        ....|....|....|....|....|....|....|....|....|....|....|....|....|....|....|....|....|....|....|....|
KFG84234 M. anisopliae E6               ------------------------GTLPD--------------------AVVFVD--------TDDLIKNVSSICHNTIVVIHSVGPVLVTDWYQNPNIS
```

**KFG86760 *M. anisopliae* E6**	-----------------------FGTIPY--------------------CIVFG----------DSLVKTVADQCNKTVVVLHNAGPRIVESFVDHPNVT
**KFG85258 *M. anisopliae* E6**	------------------------GTIPL--------------------CLVFI----------DALVNNVASKCANTIAVVHAAGIRLVDRWIEHPNVT
**KFG81708 *M. anisopliae* E6**	------------------------GTLPI--------------------ALVFIE--------YDQLVKDVASKYKNVIVVAHTVGPMILEKWIDLPAVK
**KFG84481 *M. anisopliae* E6**	-----------------------YPKVKLVD------------------SVVIIE--------KDQLITQVAAANSNTAVVMQTGTPEEM-PWLE--KTP
**ACI15900 NAGA *T. maritima* MSB8**	--
**AAK16587 CbsA *T. neapolitana***	--
**BAA32403 NAGA *S. thermoviolaceus* OPC520**	--
**AGC24356 *R. miehei* CAU432**	--
**MA128875 NAG4 *M. anisopliae* E6**	--
**XP007823900 *M. robertsii* ARSEF 23**	--
**XP007809270 *M. acridum* CQMa 102**	--
**EHK46125 *T. atroviride* IMI 206040**	--
**XP006969215 *T. reesei* QM6a**	--
**EHK20754 *T. virens* Gv29-8**	--
**XP008602406 *B. bassiana* ARSEF 2860**	--
**XP006673913 *C. militaris* CM01**	--
**XP754443 *A. fumigatus* Af293**	--
**XP001399892 *A. niger* CBS 513.88**	--
**P48823 HEXA *Alteromonas* sp. strain O7**	--
**P40406 NAGZ *B. subtilis subtilis* 168**	--
**BAC56177 NAGZ *C. paraputrificum* M21**	--
**P75949 NAGZ *E. coli* K12**	--
**P96157 NAGZ *V. furnissii* 7225**	--
**KFD82021 NAGZ *V. cholera***	--
**KFG78085 NAG3 *M. anisopliae* E6**	STGALYGFVATYFIAGVGIIGALL-----VDPSKRNVSIGRSLHRRALK-----PGVFLGIPLK------------------------------------
**XP007818395 *M. robertsii* ARSEF 23**	STGALYGFVATYFIAGVGIIGALL-----VDPSKRNVSIGRSLHRRALK-----PGVFLGIPLK------------------------------------
**XP008598947 *B. bassiana* ARSEF 2860**	STQALYGFVATYATGGVGILGALI-----VDPSKRNVSIGRSLHKRALK-----PGVFPGLALK------------------------------------
**XP006669831 *C. militaris* CM01**	STQALYGFVATYVTGAVGILGALI-----VDPTKRNVSIGRSLHKRALK-----PGVFPGLALK------------------------------------
**EHK44614 *T. atroviride* IMI 206040**	STQALYGFVATYYVQNVGMLGALV-----VDPTKRNLSIGRSLHRRALK-----PSLFLGIPLK------------------------------------
**XP006966911 *T. reesei* QM6a**	STQALYGFAATYFVQNVGILGALI-----VDPTKRNMSIGRSLHRRAIK-----PALFLGIPLK------------------------------------
**EHK23853 *T. virens* Gv29-8**	STQALYGFVATYFIQNVGILGALI-----VDPTKRNLSIGRSLHRRALK-----PALFLGIPLK------------------------------------
**XP388571 *F. graminearum* PH1**	STNALYGFAATYYIHGVGIMGGVF-----VEPTKRDVSIGRSLHRRAIR-----PGVFLGIPVK------------------------------------
**ENH61856 *F. oxysporum f.sp.cubense* race1**	STNALYGFAATYFVHGVGILGGVF-----VEPTKRDVSIGRSLHRRALR-----PGVFLGIPNK------------------------------------
**XP003053110 *N. haematococca* mpVI 77-13-4**	STGALYGFAATYFLHGVGILAAIF-----VDPSKRDVSIGRSLHRRALR-----PGVFLGIPVK------------------------------------
**XP003718403 *M. oryzae* 70-15**	STKALYGFCATYCIEGVGIIGSIF-----VDPSKRNVSVGRSLHRRALR-----PGVFLGVPIK------------------------------------
**EAA31125 *N. crassa* OR74A**	STGALYGFCSTYYLPSTGIIGAIF-----VDPSKRNLSIGYSLHRRAVR-----PGIFPGIPLK------------------------------------
**XP747213 *A. fumigatus* Af293**	STQALYGFCSTYFFRSTGVIGSLI-----VDPARRKLSIGNSLHNRAIR-----PGIYLGIP-R------------------------------------
**XP001398206 *A. niger* CBS 513.88**	STQALYGFCSTYFFRSTGVIGSLI-----VHPARRKLSIGNSLHSRAIR-----PGIYLGIP-R------------------------------------
**XP659020 *A. nidulans* FGSC A4**	TTRALYGFCSTYYFRATGVIGSLI-----VDPSRRRLSIGNSLHNRAIR-----PGIYLGIP-R------------------------------------
**Q7WUL3 NAG3 *C. fimi***	-----------------------F-----VD-----------------------EG--MAAP-V------------------------------------

```
                                               810       820       830       840       850       860       870       880       890       900
                                        ....|....|....|....|....|....|....|....|....|....|....|....|....|....|....|....|....|....|....|....|
KFG84234 M. anisopliae E6               AIVITDILYGKTS-GRSPFTWQQDFTEGSFIDYRHFDKKNSTPIYEFGHGLSWSTFEY------------------------------------------
KFG86760 M. anisopliae E6               AIILVKLLYGEAGFGKLSYTVQSNFTEGVYLDYKYFEMHNITPRYEFGFGLSYTTFSL------------------------------------------
KFG85258 M. anisopliae E6               AAILVKLLYGEANFGKLPYTLQCDFTEGVYLDYRAFDEGNVTPRYEFGYGLSYTTFSY------------------------------------------
KFG81708 M. anisopliae E6               SVLLADVLFGEVSPGHLPYSIHQDYTEGLYIDYRWLNKNNIKPRYAFGHGLSYTNFTY------------------------------------------
KFG84481 M. anisopliae E6               AVVIADILFGDANPGKLSLSF---YGEDVYMGYRYYEFANRAVNFPFGHGLSYTSFSF------------------------------------------
ACI15900 NAGA T. maritima MSB8          ----------------------------------------------------------------------------------------------------
AAK16587 CbsA T. neapolitana            ----------------------------------------------------------------------------------------------------
BAA32403 NAGA S. thermoviolaceus OPC520 ----------------------------------------------------------------------------------------------------
AGC24356 R. miehei CAU432               ----------------------------------------------------------------------------------------------------
MA128875 NAG4 M. anisopliae E6          ----------------------------------------------------------------------------------------------------
XP007823900 M. robertsii ARSEF 23       ----------------------------------------------------------------------------------------------------
XP007809270 M. acridum CQMa 102         ----------------------------------------------------------------------------------------------------
EHK46125 T. atroviride IMI 206040       ----------------------------------------------------------------------------------------------------
XP006969215 T. reesei QM6a              ----------------------------------------------------------------------------------------------------
EHK20754 T. virens Gv29-8               ----------------------------------------------------------------------------------------------------
XP008602406 B. bassiana ARSEF 2860      ----------------------------------------------------------------------------------------------------
XP006673913 C. militaris CM01           ----------------------------------------------------------------------------------------------------
XP754443 A. fumigatus Af293             ----------------------------------------------------------------------------------------------------
XP001399892 A. niger CBS 513.88         ----------------------------------------------------------------------------------------------------
P48823 HEXA Alteromonas sp. strain O7   ----------------------------------------------------------------------------------------------------
P40406 NAGZ B. subtilis subtilis 168    ----------------------------------------------------------------------------------------------------
BAC56177 NAGZ C. paraputrificum M21     ----------------------------------------------------------------------------------------------------
P75949 NAGZ E. coli K12                 ----------------------------------------------------------------------------------------------------
P96157 NAGZ V. furnissii 7225           ----------------------------------------------------------------------------------------------------
KFD82021 NAGZ V. cholera                ----------------------------------------------------------------------------------------------------
KFG78085 NAG3 M. anisopliae E6          ----------------------------------------------------------TNMIIHNLPNWVAPEGLLQSIQRANISFDLIHGLENAESVLH
XP007818395 M. robertsii ARSEF 23       ----------------------------------------------------------TNMIIHDLPNWVAPEGLLQSIQRANISFDLIHGLENAESVLH
XP008598947 B. bassiana ARSEF 2860      ----------------------------------------------------------TNMMIADLGSWTAPEGLLPTIQRANISFDLIHGLDNADTVLH
XP006669831 C. militaris CM01           ----------------------------------------------------------TNMVIADLGGWTAPEGLLPTIQRANISFDLIHGLDNAETVLH
EHK44614 T. atroviride IMI 206040       ----------------------------------------------------------TNMIIQDLSAWYAPEGLSQSIQRANISFDLIYGVESGDTVMH
XP006966911 T. reesei QM6a              ----------------------------------------------------------TNMVIQDLSAWYAPEGLSQSIQRANISFDLIYGVESGDTVMH
EHK23853 T. virens Gv29-8               ----------------------------------------------------------TNMIIHDLSAWYAPEGLSQSIQRANISFDLIYGVESGDTVMH
XP388571 F. graminearum PH1             ----------------------------------------------------------SNMVIQDLANWSAPEGLSQSIQRAGISFDLIHGLDNADGVLS
ENH61856 F. oxysporum f.sp.cubense race1----------------------------------------------------------SNMILREVASWSAPEGLSQSIQRAGISFDLIHDLDNADGVLS
XP003053110 N. haematococca mpVI 77-13-4----------------------------------------------------------TNMIIQDLPNWSAPEGLLQSIQRANISFDLIHGLDNGDGVLS
XP003718403 M. oryzae 70-15             ----------------------------------------------------------TSLAIADLPSWGAPEGLLQSIQRANISFDLIHGLESGETVLA
EAA31125 N. crassa OR74A                ----------------------------------------------------------YNLLLPSLQTWSSPPDLPTTLQLAGISFDLIPGLAEQESVLS
XP747213 A. fumigatus Af293             ----------------------------------------------------------CSVVLRNLPTWTPPDGLIQGLKNADVSYDLVHGWDYAEPILD
XP001398206 A. niger CBS 513.88         ----------------------------------------------------------CSVVLRNLTTWTPPDGLIFGLQNADVTYDLVHGWDHADSILD
XP659020 A. nidulans FGSC A4            ----------------------------------------------------------CNVALRNLQTWSPPEGLVNSLQSADAVYDLVHGWDYADSIID
Q7WUL3 NAG3 C. fimi                     -------------------------------------------------------------------EWAEPMGYVHSVPTVFVSFENPYHLD-----VP
```

```
                                                910       920       930       940       950       960       970       980       990      1000
                                         ....|....|....|....|....|....|....|....|....|....|....|....|....|....|....|....|....|....|....|....|
KFG84234 M. anisopliae E6                --------------------------------------------------------------------------STAQLWDVVYTVSATITEIPQLYISL
KFG86760 M. anisopliae E6                -----------------------------------------------------------------------------DLFDNIATVTVDVSEVPQLYVGI
KFG85258 M. anisopliae E6                -----------------------------------------------------------------------------DLWDIVARVSTTVTEVAQLYLGI
KFG81708 M. anisopliae E6                --------------------------------------------------------------------------AVKSLWDVAYKLSVVVTASVQAYLQF
KFG84481 M. anisopliae E6                --------------------------------------------------------------------------ALK------------VKEVAQMYIKP
ACI15900 NAGA T. maritima MSB8          ---------------------------------------FVLRNPFD---------------------RSTKPI--------------------------
AAK16587 CbsA T. neapolitana           ---------------------------------------FIIRNPFD---------------------HSTKPI--------------------------
BAA32403 NAGA S. thermoviolaceus OPC520 ---------------------------------------VAVRNPYDYSWTDVEVRAAARVVAGRVSPRGTVPV--------------------------
AGC24356 R. miehei CAU432               ---------------------------------------AAVMNPYDYEYTPPAHEAAVRLIFGEIETRSRLPI--------------------------
MA128875 NAG4 M. anisopliae E6           ---------------------------------------VATCSPYDYEPTLEAFASAADIIYGAATAKGRLPV--------------------------
XP007823900 M. robertsii ARSEF 23       ---------------------------------------VATCSPYDYEPTLEAFASAADIIYGAATARGTLPV--------------------------
XP007809270 M. acridum CQMa 102         ---------------------------------------VATCNPYDYEPTLEAFASAADIIYGAATARGKLPV--------------------------
EHK46125 T. atroviride IMI 206040      ---------------------------------------VATCAPYDYEPTVEAFSAAVDLLFGDAQPRGKLPV--------------------------
XP006969215 T. reesei QM6a             ---------------------------------------IATCAPYDYEPTVEAFSAAVDILFGDAQPRGKLPV--------------------------
EHK20754 T. virens Gv29-8              ---------------------------------------IATCAPYDYEPTVEAFSAAIDILFGDAQPRGKLPV--------------------------
XP008602406 B. bassiana ARSEF 2860     ---------------------------------------IATCAPYDYEPTLEAFRAAVDIIYGIDTARGKLPV--------------------------
XP006673913 C. militaris CM01          ---------------------------------------IATCAPYDYEPTLEAFRAAVDVIYDVETAKGKLPV--------------------------
XP754443 A. fumigatus Af293            ---------------------------------------IAACSPYDYEPTIEAFTAAANILFGAIIPKGALPV--------------------------
XP001399892 A. niger CBS 513.88        ---------------------------------------IAACNPYDYEPTPEAFAAAAEVIFGKSEPKGILPV--------------------------
P48823 HEXA Alteromonas sp. strain O7  ---------------------------------------ISLRAPYEYAVAGPAYTALAKVILGIAKAEGSLPV--------------------------
P40406 NAGZ B. subtilis subtilis 168   ---------------------------------------MSLRNPYDYGYLQPNIPAGVMAIFGQAKPKGTLPV--------------------------
BAC56177 NAGZ C. paraputrificum M21    ----------------------------------------------------------------------------------------------------
P75949 NAGZ E. coli K12                ----------------------------------------------------------------------------------------------------
P96157 NAGZ V. furnissii 7225          ----------------------------------------------------------------------------------------------------
KFD82021 NAGZ V. cholera              ----------------------------------------------------------------------------------------------------
KFG78085 NAG3 M. anisopliae E6        HVRANANPEVLELYTYALSENKTCGIVRAKDPSGNLIGT-----------------------------------LTY------------IP---------
XP007818395 M. robertsii ARSEF 23     HVRANANPEVLELYTYALSENKTCGIVRAKDPSGNLIGT-----------------------------------LTY------------IP---------
XP008598947 B. bassiana ARSEF 2860   HVRTESGPEVVELYRYALAETKSCGIVRAKDPSGNIMGT-----------------------------------LTY------------IP---------
XP006669831 C. militaris CM01        HVRTESGPEVVELYRYALAETKTCGIVRAKDPAGAIMGT-----------------------------------LTY------------IP---------
EHK44614 T. atroviride IMI 206040    HVRTHANPEVLELYRAALEESKACGIVRAKDAAGNLLGT-----------------------------------LRY------------VP---------
XP006966911 T. reesei QM6a           HVRTHANPEVLELYRTALEESKACGIVRAKDAAGNLLGT-----------------------------------LRY------------VP---------
EHK23853 T. virens Gv29-8            HVRTHANPEVLELYRTALEESKACGIVRAKDAAGNLLGT-----------------------------------LRY------------VP---------
XP388571 F. graminearum PH1          HVRNHASPEVLELYRHALSETKLSGIVRAKDASGNLLGT-----------------------------------LTY------------YP---------
ENH61856 F. oxysporum f.sp.cubense race1HVRNNANPEVLELYRHALSDSKLSGIVRAKDATGTLLGT-----------------------------------LTH------------IP---------
XP003053110 N. haematococca mpVI 77-13-4HVRTHANAEILELYRYALSDNKSCGIVRAKDATGNLLGT-----------------------------------LTH------------VP---------
XP003718403 M. oryzae 70-15         HVRAHANPEVQELYRAALEETKTCGVVRAKGNNDTLLGT-----------------------------------LTH------------LP---------
EAA31125 N. crassa OR74A           FVSSHSTPEILTLYRLALLQSNNTWIIRAKGGAEQVIGT-----------------------------------L-H------------LP---------
XP747213 A. fumigatus Af293          HIKTNSRQGLIDIYKIALGGAPNCGIIRATRPSDGILGT-----------------------------------LEH------------MP---------
```

```
XP001398206 A. niger CBS 513.88          HIKTNSRQGVIDIYRVALGGAPHCGIIRATRPGDGILGS-----------------------------------LEH------------MP---------
XP659020 A. nidulans FGSC A4            HVKTNSRQGVIDIYKIALGGAPHCGIIRARRPHDGILGS-----------------------------------LEH------------MP---------
Q7WUL3 NAG3 C. fimi                      RVRT---------------------LINTYGSSPVVLET-----------------------------------F-------------------------
```

```
                                               1010      1020      1030      1040      1050      1060      1070      1080      1090      1100
                                         ....|....|....|....|....|....|....|....|....|....|....|....|....|....|....|....|....|....|....|....|
KFG84234 M. anisopliae E6                PVRVLRGFNRIAPGQNVTEITRRE-------------------------------------------------------------------VITKAPKKV
KFG86760 M. anisopliae E6                PAKQLRGFEKLAPGTTVPPLTRRD-------------------------------------------------------------------RLQRGQYNI
KFG85258 M. anisopliae E6                PPKQLRGFEKLAPGQSATGLTRRD-------------------------------------------------------------------VVQRGEYGV
KFG81708 M. anisopliae E6                PIIQLRDFEKLRPGESTTQLTRKD-------------------------------------------------------------------VIPAGGYTV
KFG84481 M. anisopliae E6                PVKELRGFAKLEAGETKVELEKYV-------------------------------------------------------------------CVEAGEYEV
ACI15900 NAGA T. maritima MSB8           ----------------------------------------------------------------------------------------------------
AAK16587 CbsA T. neapolitana            ----------------------------------------------------------------------------------------------------
BAA32403 NAGA S. thermoviolaceus OPC520 ----------------------------------------------------------------------------------------------------
AGC24356 R. miehei CAU432               ----------------------------------------------------------------------------------------------------
MA128875 NAG4 M. anisopliae E6           ----------------------------------------------------------------------------------------------------
XP007823900 M. robertsii ARSEF 23       ----------------------------------------------------------------------------------------------------
XP007809270 M. acridum CQMa 102         ----------------------------------------------------------------------------------------------------
EHK46125 T. atroviride IMI 206040       ----------------------------------------------------------------------------------------------------
XP006969215 T. reesei QM6a              ----------------------------------------------------------------------------------------------------
EHK20754 T. virens Gv29-8               ----------------------------------------------------------------------------------------------------
XP008602406 B. bassiana ARSEF 2860      ----------------------------------------------------------------------------------------------------
XP006673913 C. militaris CM01           ----------------------------------------------------------------------------------------------------
XP754443 A. fumigatus Af293             ----------------------------------------------------------------------------------------------------
XP001399892 A. niger CBS 513.88         ----------------------------------------------------------------------------------------------------
P48823 HEXA Alteromonas sp. strain O7   ----------------------------------------------------------------------------------------------------
P40406 NAGZ B. subtilis subtilis 168    ----------------------------------------------------------------------------------------------------
BAC56177 NAGZ C. paraputrificum M21     ----------------------------------------------------------------------------------------------------
P75949 NAGZ E. coli K12                 ----------------------------------------------------------------------------------------------------
P96157 NAGZ V. furnissii 7225           ----------------------------------------------------------------------------------------------------
KFD82021 NAGZ V. cholera                ----------------------------------------------------------------------------------------------------
KFG78085 NAG3 M. anisopliae E6          ------------------------GIIAPIVPSPQATLVLQGLALMGVRQSKGHRASKAVLSWIVDDAFEPLVAMGFDILQAFEEITNSPE---------
XP007818395 M. robertsii ARSEF 23       ------------------------GIIAPIVPSPQATLVLQGLALMGVRQSKGHRASKAVLSWIVDDAFEPLVAMGFDILQAFEEITNSPE---------
XP008598947 B. bassiana ARSEF 2860      ------------------------GIVAPTVPPPLSTLVLQGLALMGVRQCKSHKASKAVLSWVIDDTSEPLVAMGFDALQSFEEMTNSPD---------
XP006669831 C. militaris CM01           ------------------------GIVAPTVPPPLSTLVLQGLALMGVRQCKSHKASKAVLSWVIDDTSEPLIAMGFDTLQSFEEITNSPD---------
EHK44614 T. atroviride IMI 206040       ------------------------GLLAPIVPLPLSTLVLQGLALMGVRQNKGHKANKTVLSWVVDDAFEPLVAMGFDVLQAFEEITNSPE---------
XP006966911 T. reesei QM6a              ------------------------GLLAPIVPPPLSTLVLQGLALMGVRQNKGHKATKSVLSWVVDDAYEPLVAMGFDVLQAFEEITNSPE---------
EHK23853 T. virens Gv29-8               ------------------------GLLAPIVPPPLSTLVLQGLALMGVRQNKGHKATQSVLSWVVDDAFEPLVAMGFDVLQAFEEITNSPE---------
```

```
XP388571 F. graminearum PH1             ------------------------GILAPVVPSPLATLTLQGLAFMGVRQARNHKATKVMLGSSVDDGTESLTAMGFETLQAFEEITNSPE---------
ENH61856 F. oxysporum f.sp.cubense race1------------------------GIIAPVVPPPQTTLVLQGLTLMGIRQARSHKATKVVLGCVVDD-TESLTAMGFETLQEFEEITNSPE---------
XP003053110 N. haematococca mpVI 77-13-4------------------------GILAPIVPAPQSNLVLQGLALMGVRQSKGHKAAKVVLGWVVDDGTEPLAAMGFESLQAFEEITNSPE---------
XP003718403 M. oryzae 70-15             ------------------------GIIAPVLAPHSGVLVLQGLALMGLRQNKAHKSSRSVLSWVYDDAFEPLVAMGFEIAQQFEEITNSLD---------
EAA31125 N. crassa OR74A                ------------------------GIIAPVVSSQQQQLVLQGLVMLGLRQNKREKMACCLLSWVSNNSVETLLGMGFEVQQVWEEVVNGVE---------
XP747213 A. fumigatus Af293             ------------------------GISSPVISPEYAT-VMQGLVLLGIKQIRRQGAEAVVIDCVGDSNFDCLSGMGFSMLHSFEEVNCDAA---------
XP001398206 A. niger CBS 513.88         ------------------------GISSPVISPEYAT-VMQGLVLLGIKQIRKQGAEAVIMDSVVDGNFECLSGLGFSTLHSFEEVNCDAA---------
XP659020 A. nidulans FGSC A4            ------------------------GISSPVIWPEYAT-LLQGLILLGIKQIRRQGADAVVIDCVVDSNFDWLTEIGFTTLHSYEEVNCDAA---------
Q7WUL3 NAG3 C. fimi                     ----------------------------------------------------------------------------------------------------
```

```
                                        ....|...
KFG84234 M. anisopliae E6               FVGSSSRN
KFG86760 M. anisopliae E6               YVGTSSRD
KFG85258 M. anisopliae E6               FVGASSRD
KFG81708 M. anisopliae E6               WLGGASDD
KFG84481 M. anisopliae E6               IISDSSRD
ACI15900 NAGA T. maritima MSB8          --------
AAK16587 CbsA T. neapolitana            --------
BAA32403 NAGA S. thermoviolaceus OPC520 --------
AGC24356 R. miehei CAU432               --------
MA128875 NAG4 M. anisopliae E6          --------
XP007823900 M. robertsii ARSEF 23       --------
XP007809270 M. acridum CQMa 102         --------
EHK46125 T. atroviride IMI 206040       --------
XP006969215 T. reesei QM6a              --------
EHK20754 T. virens Gv29-8               --------
XP008602406 B. bassiana ARSEF 2860      --------
XP006673913 C. militaris CM01           --------
XP754443 A. fumigatus Af293             --------
XP001399892 A. niger CBS 513.88         --------
P48823 HEXA Alteromonas sp. strain O7   --------
P40406 NAGZ B. subtilis subtilis 168    --------
BAC56177 NAGZ C. paraputrificum M21     --------
P75949 NAGZ E. coli K12                 --------
P96157 NAGZ V. furnissii 7225           --------
KFD82021 NAGZ V. cholera                --------
KFG78085 NAG3 M. anisopliae E6          --------
```

```
XP007818395 M. robertsii ARSEF 23       --------
XP008598947 B. bassiana ARSEF 2860      --------
XP006669831 C. militaris CM01           --------
EHK44614 T. atroviride IMI 206040       --------
XP006966911 T. reesei QM6a              --------
EHK23853 T. virens Gv29-8               --------
XP388571 F. graminearum PH1             --------
ENH61856 F. oxysporum f.sp.cubense race1--------
XP003053110 N. haematococca mpVI 77-13-4--------
XP003718403 M. oryzae 70-15             --------
EAA31125 N. crassa OR74A                --------
XP747213 A. fumigatus Af293             --------
XP001398206 A. niger CBS 513.88         --------
XP659020 A. nidulans FGSC A4            --------
Q7WUL3 NAG3 C. fimi                     --------
```

Figure S4 - Multiple alignment of GH3 NAGases from bacteria, zygomycetes, filamentous fungi, and *M. anisopliae* β-glucosidases. Amino acid alignment built and trimmed with GUIDANCE2 using PRANK as the MSA algorithm with 100 bootstrap replicates. Location of the GH20 conserved sequence motif (K-H-F/I-P-G-H/L-G-x-x-x-x-D-S/T-H) is highlighted.
