# Supplementary material for: Molecular evolution and transcriptional profile of GH3 and GH20 β-N-acetylglucosaminidases in the entomopathogenic fungus Metarhizium anisopliae
Source: Genet Mol Biol. 2018 Dec 10;41(4):843–57. doi: 10.1590/1678-4685-GMB-2017-0363 (PMC6415606; doi:10.1590/1678-4685-GMB-2017-0363)
Supplement: Supplementary file 5 [file 1415-4757-GMB-1678-4685-GMB-2017-0363-s005.pdf]

Supplementary Material to "Molecular evolution and transcriptional profile of GH3 and GH20 β-N-acetylglucosaminidases in the entomopathogenic fungus Metarhizium anisopliae"

Table S1 - Primer sequences used in qPCR to target *M. anisopliae* GH20 and GH3 NAGase genes and reference gene.

Acronym	Gene description	Accession number	Forward/Reverse primer sequences (5'-3')
NAG1	putative NAGase GH20	KFG80340	TCCAGCAACTCGTCATCTTC / AGGCAAGTCCGTCAATCTG
NAG2	putative NAGase GH20	KFG85702	CGGCGAGACCAAAGAGTC / CATCCACGGCACAAGAC
NAG3	putative NAGase GH3	KFG78085	CCGAATGTCTGGAAATGGAGGC / CGTCTGATGGATGTGAAGATGCG
NAG4	putative NAGase GH3	MANI128875	TCTGTCCCCCGAGACGATCAAG / CATGACATTGTCGACGCCCG
TEF1α	Translation elongation factor 1-α	AY445082	CGGCAAGTCTACCACCACTG / TGATACCACGCTCACGCTC
